# Oxidative stress activates NORAD expression by H3K27ac and promotes oxaliplatin resistance in gastric cancer by enhancing autophagy flux via targeting the miR-433-3p

**DOI:** 10.1038/s41419-020-03368-y

**Published:** 2021-01-18

**Authors:** Jizhao Wang, Yuchen Sun, Xing Zhang, Hui Cai, Cheng Zhang, Hangying Qu, Lin Liu, Mingxin Zhang, Junke Fu, Jia Zhang, Jiansheng Wang, Guangjian Zhang

**Affiliations:** 1 Department of Thoracic Surgery, The First Affiliated Hospital of Xi’an Jiaotong University, Xi’an, Shaanxi China; 2 Department of Radiation Oncology, The First Affiliated Hospital of Xi’an Jiaotong University, Xi’an, Shaanxi China; 3 Department of Hepatology Surgery, The First Affiliated Hospital of Xi’an Jiaotong University, Xi’an, Shaanxi China; 4 Department of Vascular Surgery, The First Affiliated Hospital of Xi’an Jiaotong University, Xi’an, Shaanxi China; 5 Department of Gastroenterology, The First Affiliated Hospital of Xi’an medical University, Xi’an, Shaanxi China

**Keywords:** Gastric cancer, Oncogenes

## Abstract

Oxaliplatin resistance undermines its curative effects on cancer and usually leads to local recurrence. The oxidative stress induced DNA damage repair response is an important mechanism for inducing oxaliplatin resistance by activating autophagy. ELISA is used to detect target genes expression. TMT-based quantitative proteomic analysis was used to investigate the potential mechanisms involved in NORAD interactions based on GO analysis. Transwell assays and apoptosis flow cytometry were used for biological function analysis. CCK-8 was used to calculate IC50 and resistance index (RI) values. Dual-luciferase reporter gene assay, RIP and ChIP assays, and RNA pull-down were used to detect the interaction. Autophagy flux was evaluated using electron microscope and western blotting. Oxidative stress was enhanced by oxaliplatin; and oxaliplatin resistance gastric cancer cell showed lower oxidative stress. TMT labeling showed that NORAD may regulate autophagy flux. NORAD was highly expressed in oxaliplatin-resistant tissues. In vitro experiments indicate that NORAD knockdown decreases the RI (Resistance Index). Oxaliplatin induces oxidative stress and upregulates the expression of NORAD. SGC-7901 shows enhanced oxidative stress than oxaliplatin-resistant cells (SGC-7901-R). NORAD, activated by H3K27ac and CREBBP, enhanced the autophagy flux in SGC-7901-R to suppress the oxidative stress. NORAD binds to miR-433-3p and thereby stabilize the ATG5- ATG12 complex. Our findings illustrate that NORAD, activated by the oxidative stress, can positively regulate ATG5 and ATG12 and enhance the autophagy flux by sponging miR-433-3p. NORAD may be a potential biomarker for predicting oxaliplatin resistance and mediating oxidative stress, and provides therapeutic targets for reversing oxaliplatin resistance.

## Graphical abstract

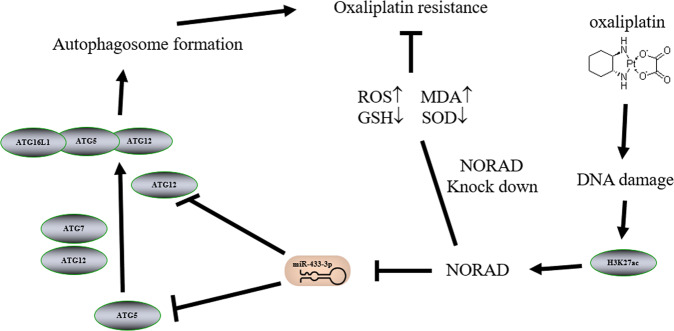

## Background

Gastric cancer is among the most lethal cancers worldwide, and has a median recurrence time of 11.7 months^1^. The poor prognosis is due to the tendency to recurrence and the poor therapeutic effects of chemotherapy^2^. Platinum-based chemotherapy, such as that with XELOX (oxaliplatin and capecitabine), can significantly improve the survival rate and control local recurrence^3^. Additionally, oxaliplatin-containing regimens may be superior to other platinum-containing regimens^4^. For example, oxaliplatin can prolong less-than-a-month overall survival compared with cisplatin^5^. In clinical practice, resistance to oxaliplatin usually leads to the failure of chemotherapy^6^. Therefore, in this study, we aimed to investigate the key regulatory genes involved in modulating oxaliplatin resistance.

Oxaliplatin exerts its anticancer effects mainly through binding to and damaging DNA, thereby inhibiting DNA replication^7^. Oxaliplatin-induced DNA damage repair occurs primarily through the nucleotide excision repair (NER) pathway, the activation of which normally leads to oxaliplatin resistance^8^. In addition to NER, autophagy is also reported to be involved in regulating oxaliplatin resistance. For example, Ren et al. showed that autophagy activation induces oxaliplatin resistance in hepatocellular carcinoma, which can be reversed by miR-125b^9^. Moreover, autophagy activated by HMGB1 was also shown to play an important role in decreasing oxaliplatin sensitivity in colorectal cancer^10^. The ATG5-ATG12-ATG16 complex, which possesses E3 ubiquitin-like activity, is crucial for autophagosome formation. ATG16 mediates the localization of the complex to the phagophore assembly site and the ATG5-ATG12 conjugate catalyzes the covalent attachment of LC3 to PE^11^. Additionally, ATG7, an E1 ubiquitin-like enzyme, and ATG10, an E2 ubiquitin-like enzyme, are required for the initial conjugation of ATG5 and ATG12^12^. However, whether autophagy is involved in oxaliplatin resistance in gastric cancer is unknown. Several studies have indicated that autophagy can be induced by the oxaliplatin-related DNA damage response, which then promotes oxaliplatin resistance^13,14^.

Oxidative stress has been found to be associated to generate DNA damage and induce apoptosis^15^. To date, accumulating evidences have indicated the essential role of oxidative stress in mediating platinum resistance. Sen Wang et al. illustrated that PRDX2 induced by H.pylori infection suppressed oxidative stress and double-strand breaks to promote cisplatin resistance^16^. Besides, reactive oxygen species production can be increased by cannabidiol, resulting in enhanced autophagy and sensitizing colorectal cancer cells to oxaliplatin^14^. In this context, we assumed the essential role of oxidative stress in mediating oxaliplatin resistance by regulating DNA damage response and autophagy.

Increasing evidence has indicated that microRNAs (miRNAs) and long noncoding RNAs (lncRNAs) are important for mediating multidrug resistance^17^. NORAD was identified and named by Sungyul et al., who reported that NORAD (noncoding RNA activated by DNA damage) was induced by DNA damage and showed strong regulatory activity towards Pumilio-Fem3-binding factor (PUF), which regulates downstream target mRNAs by activating the deadenylating and decapping process^18^. Our group previously reported that NORAD can be induced by radiation, indicating its potential involvement in DNA damage responses^19^. Moreover, accumulating evidence has indicated that NORAD mostly resides in the cytoplasm, and that NORAD may act as a target miRNA sponge and reverse their biological functions, including for miR-656-3p, miR-615-3p, and miR-202-5p^19–21^. Analysis using the UCSC genome browser led us to predict that H3K27ac may be enriched at the NORAD promoter and act as an important regulator of NORAD expression^22^. Histone H3 lysine 27 acetylation (H3K27ac) is usually found at transcription start sites, and interacts with active enhancers to promote gene expression^23^. CREB-binding protein (CBP) and P300, acting as the acetyltransferase, account for the H3K27ac in the promoter region of target genes to exert their transcriptional coactivator function^24,25^. A previous study reported that H3K27ac regulates the expression of GLI1, which is induced by cisplatin in a dose-dependent manner^26^. Therefore, we hypothesized that oxaliplatin-related DNA damage responses may upregulate the levels of H3K27ac, leading to the overexpression of NORAD and consequently oxaliplatin resistance.

In this study, using online bioinformatics tool, Starbase V2.0, we identified that miR-433-3p may be associated with oxaliplatin resistance^27^. MiR-433-3p is a potential tumor suppressor in esophageal cancer and glioma^28,29^. Importantly, miR-433-3p can enhance the sensitivity of glioma cells to temozolomide, which also induces DNA damage^29^. We also noticed that miR-433-3p is predicted to bind to the 3′-UTR of *ATG5* and *ATG12*^27^. This suggested that NORAD could compete with miR-433-3p for binding to ATG5 and ATG12, thereby enhancing the autophagy flux and promoting oxaliplatin resistance.

## Methods

### Cell culture, transfection, and transduction

The SGC-7901 and KATO III cell lines were purchased and authenticated from the Cell Bank of the Chinese Academy of Sciences Typical Culture Preservation Committee (Shanghai, China); The GES-1 cell line was purchased from American Type Culture Collection (ATCC, Manassas, USA). SGC-7901, KATO III, and GES-1 were cultured in 1640-medium supplemented with 10% fetal bovine serum (FBS; ThermoFisher Scientific, Shanghai, China) at 37 °C and 5% CO_2_.

Lentiviral constructs for NORAD and miR-433-3p knockdown, miR-433-3p overexpression, and respective negative controls (NCs) were synthesized by Genechem (Shanghai, China). Lentiviral constructs were transduced into target cells at the concentration of 1 × 10^8^ TU/mL with transduction enhancement P solution. hU6-MCSUbiquitin-EGFP-IRES-puromycin was used as the vector for the miR-433-3p overexpression lentivirus; hU6-MCSCMV-EGFP was used as the vector for the NORAD and miR-433-3p knockdown lentiviruses. The NORAD knockdown plasmid, NORAD overexpression plasmid containing the wild-type binding site for miR-433-3p, NORAD overexpression plasmid containing a mutated binding site for miR-433-3p, miR-433-3p overexpression plasmid, and miR-433-3p knockdown plasmid were designed and synthesized by Genechem. The plasmid vector used was CMV-MCS-SV4-Neomycin. Cells were transfected with the above plasmids using Lipofectamine 3000 (Thermo Fisher Scientific, Shanghai, China) for analysis of autophagy and apoptosis.

### Patients

Our group has recruited 379 T1b-T3 stage gastric cancer patients, who were pathologically diagnosed from 2013 January to 2015 December in First affiliated hospital of Xi’an Jiaotong Unversity and First affiliated hospital of Xi’an medical hospital. Our study has been approved by ethnics committee of First affiliated hospital of Xi’an Jiaotong Unversity and ethnics committee of First affiliated hospital of Xi’an medical hospital. Our study has been carried out according to the declaration of Helsinki. Ever patient recruited was informed and signed the consent for acquiring their tissues.

### Whole-genome and miRNA sequencing chips

Twenty GeneChip PrimeView Human gene expression arrays (100-format, Affymetrix) were used for 10 gastric cancer tissues and corresponding adjacent normal tissues. The Agilent RNA 6000 Nano Kit was used for RNA quality control and the GeneChip 3′ IVT PLUS Kit was used for in vitro transcription. The GeneChip Hybridization Wash and Stain Kit was used for chip hybridization, washing, and staining. Finally, chips were scanned by GeneChip Scanner 3000. The above process was assisted, advised, and supervised by Genechem. For miRNAs, 20 GeneChip miRNA 4.0 chips (100-format, Affymetrix) were used. The Agilent RNA 6000 Nano Kit was used for RNA quality control. The FlashTag Biotin HSR RNA Labeling Kit was used for miRNA labeling. Chip hybridization, washing, staining, and scanning was as for the whole-genome chip process. The above process was assisted, advised, and supervised by Genechem. TCGA and GTEx gastric cancer patient data were also included to confirm the result of our whole-genome sequencing data analysis.

### Quantitative analysis of TMT (Tandem Mass Tag)-labeled proteins

SGC-7901 cells were used as controls, and NORAD knockdown SGC-7901 cells as the experimental group. SDT lysis buffer was used for protein extraction. Extracted protein (20 μg) was electrophoresed on 12% SDS–PAGE at 220 V for 40 min. FASP enzymolysis was then carried out with trypsin buffer; a C18 Cartridge was used for desalination of peptide fragments. The Easy nLC system was used for chromatography. The whole process was assisted, advised, and supervised by Genechem.

### RNA extraction and qRT-PCR

TRIzol reagent (Invitrogen, Carlsbad, CA, USA) was used for total RNA extraction. The PrimeScript™ RT reagent Kit (TaKaRa, Dalian, China) was used for reverse transcription. SYBR Premix Ex TaqTM II (TaKaRa) was used for qPCR. The NucleoSpin miRNA kit (TaKaRa) was used for purification of the RNA used for miRNA qRT-PCR. The purified RNA was then treated with DNase I if the RNA was extracted from transfected cells. The Mir-X miRNA qRT-PCR TB Green Kit (TaKaRa) was used for reverse transcription and qPCR. *GAPDH* was used as the control for NORAD, and *U6* for miR-433-3p. The following primers were used: GAPDH, forward: 5′-TGCCAAATATGACATCAAGAA-3′ and reverse: 5′-GGAGTGGGTGTCGTCGCTGTTG-3′; NORAD, forward: 5′-AAGCTGCTCTCAACTCCACC-3′ and reverse: 5′-GGACGTATCGCTTCCAGAGG-3′; and miR-433-3p, forward: 5′-CGATCATGATGGGCTCCTCG-3′ and reverse: 5′-GTGCAGGGTCCGAGGT-3′.

### Protein extraction and western blot

Total protein was extracted using RIPA buffer (Sigma–Aldrich, Cambridge, MA, USA), which was then validated by BCA (Sigma–Aldrich, Cambridge, MA, USA). Proteins were electrophoresed on 10% SDS–PAGE gels and then transferred onto PVDF membranes. The membranes were blocked with 5% nonfat milk in TBS-T for 2 h at room temperature. Primary antibodies were incubated overnight at 4 °C. The following day, secondary antibodies were incubated for 1 h at room temperature.

### Analysis of autophagy

SensGFP-StubRFP-LC3 lentivirus, which was constructed by Genechem, were used to transfect target cells. After 24 h of transfection, puromycin was used to eliminate unaffected cells for 3 days. Then cells were resuspended and plated into 96-well plates at the concentration of 1 × 10^4^/well for 18 h. Confocal Quantitative Image cytometer (YOKOGAWA, Tokyo, Japan) was used to scan and analyze the cells.

### Construction of the oxaliplatin-resistant cell line

The oxaliplatin-resistant SGC-7901 and KATO III cell line were named SGC-7901-R and KATO III-R respectively. First, SGC-7901 and KATO III cells were incubated with 2 µg/mL oxaliplatin for 24 h; then, the medium was changed to oxaliplatin-free medium and cell growth was observed. The above process was repeated several times until SGC-7901 and KATO III cells overcame inhibition of proliferation. The oxaliplatin concentration was then increased to 3, 4, 6, 8, and 10 µg/mL. Finally, SGC-7901 and KATO III cells that survived with 10 µg/mL oxaliplatin were regarded as oxaliplatin-resistant (KATO III-R and SGC-7901-R).

### IC50 and resistance index (RI)

Cell counting kit-8 (CCK-8, Apexbio, USA) was used to evaluate the viability of target cells. Cells (1 × 10^5^) were added to each well and culture medium containing 1, 2, 4, 8, 16, 32, and 64 μg/mL oxaliplatin was added to individual wells. After culturing for 48 h, 10 µl of the CCK-8 solution was added to each well and incubated for 2 h. The absorbance at 490 nm was measured for each well. The IC50 was calculated using GraphPad Prism 8.2 (GraphPad Software, La Jolla, CA, USA). The RI was calculated as IC50 of Resistant cells/IC50 of Parental cells.

### Evaluation of oxidative stress

Superoxide Dismutase (SOD) assay kit, Malondialdehyde (MDA) assay kit and Glutathione Peroxidase (GSH-PX) assay kit were used to detect the level of SOD, MDA, and GSH in target cells and tissues according to manufactures’ guides.

### ROS production

DHE (Dihydroethidium) Assay Kit—Reactive Oxygen Species (ab236206, Abcam China, Shanghai, China) was purchased to measure the ROS level in live target cells. Total DHE fluorescence intensity represents the ROS generation. Around 1 × 10^5^ cells were resuspended and added to V-bottom plate, which was centrifuged at 400 × *g*. Then 130 μL ROS staining buffer and 100 μL Cell-Based Assay Buffer. The fluorescence intensity was measured using 480 nm excitation wavelength and 570 nm emission wavelength.

### ChIP assay

The EZ-Magna ChIP™ A/G Chromatin Immunoprecipitation Kit (Sigma–Aldrich, Darmstadt, Germany) was used for the assay. Target cells were treated with formaldehyde to ensure the co-precipitation of DNA with proteins. The cells were then sonicated to generate 200–500 bp DNA fragments, which were validated by electrophoresis. Subsequently, the generated DNA fragments were immunoprecipitated with anti-H3K27ac and anti-CBP antibodies. An IgG antibody was used as the control. qPCR was used to detect NORAD expression.

### RIP assay

Magna RIP™ RNA-Binding Protein Immunoprecipitation Kit (Millipore, Billerica, MA, USA) was used for RIP assay. RIP lysis buffer was used for lysing target cells and then Ago2 antibody was used for immunoprecipitation; IgG antibody was used for control. The whole process was assisted, advised and supervised by Genecreate(Wuhan, China).

### FISH

Cell slides were pretreated with HCL for 20 min, the 1% NaSCN at 80 °C for 30 min, and 4% pepsin at 37 °C for 10 min. The cells were then fixed in neutral formalin at room temperature for 10 min. Then, the cells were treated with a prehybridization solution (50% deionized formamide, 5 × SSC, 5 × Denhardt, 0.02% SDS, 0.1 mg/mL yeast tRNA, 100 µg/mL denatured and sheared salmon sperm DNA) at 50 °C for 2 h. The cells were then hybridized with a NORAD probe at 48 °C for 6 h. The cells were subsequently washed with 2 × SSC and 0.3% NP-40 at 72 °C for 30 min and stained with DAPI for 5 min. The cells were observed under a confocal microscope.

### Dual-luciferase reporter assay

NORAD overexpression plasmids containing either the wild-type NORAD binding site (NORAD-WT) or a mutated NORAD binding site (NORAD-Mut) for miR-433-3p and miR-433-3p overexpression plasmids were constructed by Genechem using GV272. The negative control vector was SV40-firefly_Luciferase-MCS. Cells were co-transfected with NORAD and miR-433-3p overexpression plasmids, as follows: NORAD-WT + miR-433-3p, NORAD-Mut + miR-433-3p, NORAD-WT + NC, and NORAD-Mut + NC. After 48 h, firefly and *Renilla* luciferase activities were measured.

### RNA pull-down assay

The biotinylated probes miR-433-3p-Wt and miR-433-3p-Mut were synthesized by Genecreate (Wuhan, China). The probe sequence for miR-433-3p-WT was AUCAUGAUGGGCUCCUCGGUGU and for miR-433-3p-Mut the sequence was AAGUACUAGGGCUCCUCGGUGU. Target cells were transfected with the above probes. After 48 h, the biotinylated RNA was bound to M‐280 Streptavidin-coated MagneSphere particles using RNase‐free bovine serum albumin (BSA) and yeast tRNA. After elution, RNA was combined, harvested, and purified. The RNA was then reverse-transcribed into cDNA and qPCR was used to evaluate NORAD enrichment.

### Apoptosis

NORAD knockdown plasmids were used for transfection. The FITC Annexin V Apoptosis Detection Kit I (BD Pharmingen TM, New Jersey, USA) was used for apoptosis testing. Target cells were stained with Annexin V–FITC at room temperature for 15 min, followed by flow cytometry to detect fluorescence intensity.

### Transwell assay

Cells (5 × 10^5^) were plated into the upper chamber of Transwell plates for migration tests; chambers with Matrigel-coated membranes were used for invasion tests. Culture medium supplemented with 20% FBS was added to the lower chambers. Cells were then cultured in serum-free medium in the upper chamber for 24 h at 37 °C and 5% CO_2_. Cells from the upper chambers were gently wiped off and those from the lower chambers were stained with crystal violet and counted under an optical microscope.

### Animal model

We have recruited 20 nude mice (20–25 g, 4 weeks old) in this study. The mice were purchased form Animal center of Xi’an Jiaotong University. The 20 nude mice were then grouped into four groups. No blinding was carried out when grouping the mice. 1 × 10^6^ SGC-7901, SGC-7901-R, and SGC-7901-R NORAD knockdown and relevant NC cells were injected into the right flank of mice to produce xenograft tumors. When the volume of xenograft tumors reached ~300–400 mm^3^, we began treating mice with 10 μg/kg oxaliplatin every week for 2 weeks through tail vein injection. After 4 weeks, xenograft tumors were harvested for further analysis.

### Statistics

Data analysis was performed using R 3.3.1. GraphPad Prism 8.2 was used for graphing. ImageJ was used to quantify the western blotting and PCR results. Data are presented as means ± standard deviation. Paired *t*-tests were used for comparison between two groups. The R package WGCNA was used to perform WGCN analysis. The R package randomForest was used to construct the random-forest model.

## Results

### Oxidative stress plays an important part in mediating oxaliplatin resistance of gastric cancer cell

**Fig. 1:**
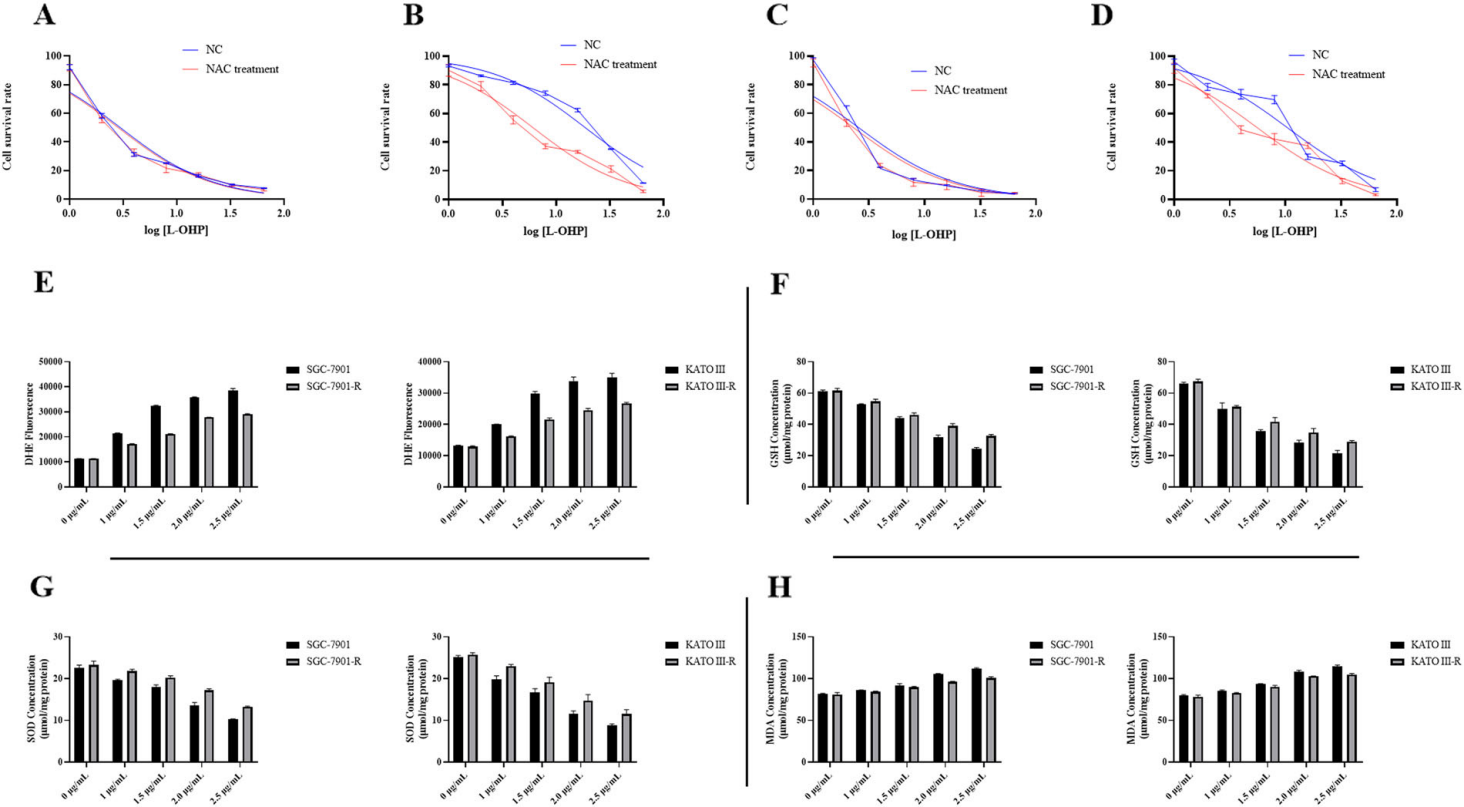
Oxidative stress in oxaliplatin resistant gastric cancer cells. **A** CCK-8 test for SGC-7901 to evaluate the IC50 after treating with or without NAC. Two way ANOVA test, NC vs NAC: 1 μg/ml L-OHP, *t* = 0.208, På 0.999; 2 μg/ml L-OHP, *t* = 2.914, *p* = 0.033, 4 μg/ml L-OHP, *t* = 1.249, *P* = 0.818; 8 μg/ml L-OHP, *t* = 3.642, *P* = 0.004; 16 μg/ml L-OHP, *t* = 0.936, *P* = 0.9521; 32 μg/ml L-OHP, *t* = 0.312, På 0.999; 64 μg/ml L-OHP, *t* = 1.041, *P* = 0.919. **B** CCK-8 test for SGC-7901-R to evaluate the IC50 after treating with or without NAC. Two way ANOVA test, NC vs NAC: 1 μg/ml L-OHP, *t* = 2.649, *P* = 0.068; 2 μg/ml L-OHP, *t* = 6.519, *P* < 0.000, 4 μg/ml L-OHP, *t* = 23.26, *P* < 0.000; 4 μg/ml L-OHP, *t* = 33.32, *P* < 0.000; 16 μg/ml L-OHP, *t* = 26.14, *P* < 0.000; 32 μg/ml L-OHP, *t* = 12.58, *P* < 0.000; 64 μg/ml L-OHP, *t* = 5.127, *P* < 0.000. **C** CCK-8 test for KATO III to evaluate the IC50 after treating with or without NAC. Two way ANOVA test, NC vs NAC: 1 μg/ml L-OHP, *t* = 1.998, *P* = 0.299; 2 μg/ml L-OHP, *t* = 9.172, *P* < 0.000, 4 μg/ml L-OHP, *t* = 1.816, *P* = 0.414; 8 μg/ml L-OHP, *t* = 1.544, *P* = 0.614; 16 μg/ml L-OHP, *t* = 0.817, *P* = 0.977; 32 μg/ml L-OHP, *t* = 0.999, *P* = 0.934; 64 μg/ml L-OHP, *t* = 0.545, *P* < 0.000. **D** CCK-8 test for KATO III-R to evaluate the IC50 after treating with or without NAC. Two way ANOVA test, NC vs NAC: 1 μg/ml L-OHP, *t* = 3.396, *P* = 0.008; 2 μg/ml L-OHP, *t* = 4.553, *P* < 0.000, 4 μg/ml L-OHP, *t* = 17.92, *P* < 0.000; 8 μg/ml L-OHP, *t* = 19.94, *P* < 0.000; 16 μg/ml L-OHP, *t* = 5.492, *P* < 0.000; 32 μg/ml L-OHP, *t* = 8.961, *P* < 0.000; 64 μg/ml L-OHP, *t* = 2.602, *P* = 0.077. **E** DHE method to test the ROS production in SGC-7901, SGC-7901-R, KATO III, and KATO III-R when treated with 0, 1, 1.5, 2.0, 2.5 μg/ml L-OHP. Tukey’s multiple comparisons test was used to evaluate the statistical significance of DHE treated with different concentration of L-OHP. SGC-7901, SGC-7901-R, KATO III, KATO III-R: 0 μg/ml vs 1 μg/ml, *P* < 0.000; 0 μg/ml vs 1.5 μg/ml, *P* < 0.000; 0 μg/ml vs 2.0 μg/ml, *P* < 0.000; 0 μg/ml vs 2.5 μg/ml, *P* < 0.000. **F** GSH expression in SGC-7901, SGC-7901-R, KATO III and KATO III-R when treated with 0, 1, 1.5, 2.0, 2.5 μg/ml L-OHP. Tukey’s multiple comparisons test was used to evaluate the statistical significance of GSH expression treated with different concentration of L-OHP. SGC-7901, SGC-7901-R, KATO III, KATO III-R: 0 μg/ml vs 1 μg/ml, *P* < 0.000; 0 μg/ml vs 1.5 μg/ml, *P* < 0.000; 0 μg/ml vs 2.0 μg/ml, *P* < 0.000; 0 μg/ml vs 2.5 μg/ml, *P* < 0.000. **G** SOD expression in SGC-7901, SGC-7901-R, KATO III, and KATO III-R when treated with 0, 1, 1.5, 2.0, 2.5 μg/ml L-OHP. Tukey’s multiple comparisons test was used to evaluate the statistical significance of SOD expression treated with different concentration of L-OHP. SGC-7901, SGC-7901-R, KATO III, KATO III-R: 0 μg/ml vs 1 μg/ml, *P* < 0.000; 0 μg/ml vs 1.5 μg/ml, *P* < 0.000; 0 μg/ml vs 2.0 μg/ml, *P* < 0.000; 0 μg/ml vs 2.5 μg/ml, *P* < 0.000. **H** MDA expression in SGC-7901, SGC-7901-R, KATO III, and KATO III-R when treated with 0, 1, 1.5, 2.0, 2.5 μg/ml L-OHP. Tukey’s multiple comparisons test was used to evaluate the statistical significance of MDA expression treated with different concentration of L-OHP. SGC-7901, SGC-7901-R, KATO III, KATO III-R: 0 μg/ml vs 1 μg/ml, *P* < 0.000; 0 μg/ml vs 1.5 μg/ml, *P* < 0.000; 0 μg/ml vs 2.0 μg/ml, *P* < 0.000; 0 μg/ml vs 2.5 μg/ml, *P* < 0.000.

We generated oxaliplatin-resistant SGC-7901 and KATO III cell lines, which we named SGC-7901-R and KATO III-R. The IC50 value for SGC-7901 cells was 2.97 μg/mL (Fig. 1A), while for the SGC-7901-R cells, the IC50 was 18.73 μg/mL (Fig. 1B). This resulted in a RI(Resistance index) of 6.31 (18.73/2.97), indicating that SGC-7901-R cells were oxaliplatin resistant. Besides, after treated with 5 mM NAC, the IC50 value for SGC-7901 and SGC-7901-R were 2.84 and 6.11 μg/mL respectively; and the RI was 2.15 (6.11/2.84), which was significantly decreased compared with 6.31. The IC50 value for KATO III and KATO III-R were 2.58 and 10.45 μg/mL respectively, the RI was 4.05 (10.45/2.58); and 5 mM NAC significantly decreased the RI (5.60/2.30 = 2.43) as well (Fig. 1C, D).

When treated with oxaliplatin, the ROS production in gastric cancer cells was significantly enhanced (Fig. 1E); besides GSH and SOD were decreased and MDA level was increased (Fig. 1F–H). Moreover, we noticed that ROS production and MDA were lower and GSH and SOD level were higher in oxaliplatin-resistant cells than that in their parental cells (Fig. 1E–H).

### NORAD was associated with DNA damage and autophagy in gastric cancer cell

**Fig. 2:**
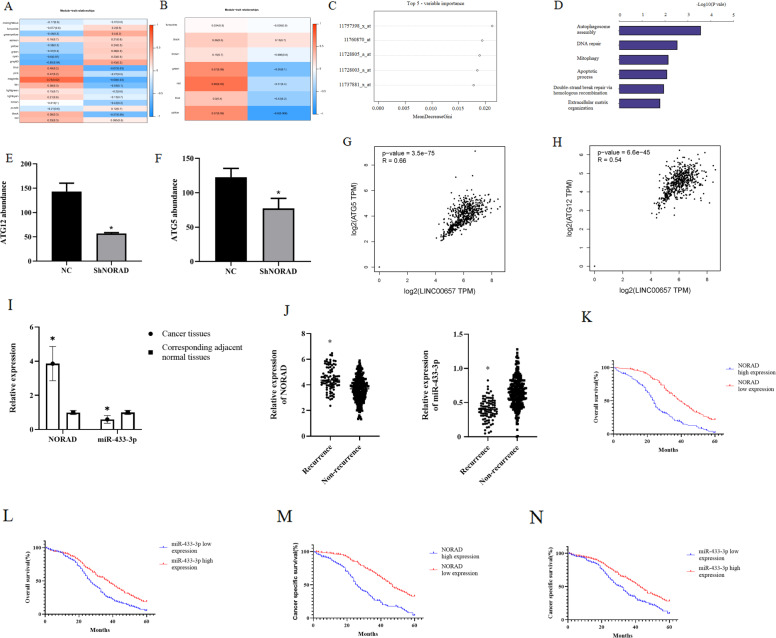
NORAD was identified to be involved in DNA damage and autophagy. **A** WGCNA for the relationship between gene modules and clinical traits. **B** WGCNA for the relationship between microRNA modules and clinical traits. **C** Top five genes ranked by importance based on random-forest model. **D** GO biological process pathways involved for differentially expressed genes after NORAD knockdown using TMT-based quantitative proteomic analysis in SGC-7901 and SGC-7901-R cell lines. **E** The influence of NORAD knockdown on ATG12 abundance using TMT-based quantitative proteomic analysis between SGC-7901 and SGC-7901-R cell line (*t* = 6.721, *P* < 0.000). **F** The influence of NORAD knockdown on ATG5 abundance using TMT-based quantitative proteomic analysis between SGC-7901 and SGC-7901-R cell line (*t* = 4.032, *P* < 0.000). **G** The association between NORAD and ATG5 based on TCGA and GTEx data. **H** The association between NORAD and ATG12 based on TCGA and GTEx data. **I** The expression of NORAD (*t* = 6.538, *P* < 0.000) and miR-433-3p (*t* = 4.276, *P* < 0.000) in 379 gastric cancer patients. **J** The expression profile of NORAD (*t* = 2.817, *P* = 0.003) and miR-433-3p (*t* = 2.175, *P* = 0.008) in nonrecurrent and recurrent gastric cancer patients. **K**–**N** The overall survival and cancer-specific survival for NORAD (OS, χ^2^ = 65.28, *P* < 0.000, 2K; CSS, χ^2^ = 61.06, *P* < 0.000, 2M) and miR-433-3p (OS, χ^2^ = 20.56, *P* < 0.000, 2L; CSS, χ^2^ = 16.92, *P* < 0.000, 2N) in 379 gastric cancer patients.

We recruited 10 gastric cancer patients who had been subjected to XELOX treatment. To identify XELOX resistance-associated genes and miRNAs, we used weighted gene co-expression network analysis (WGCNA) to group them into several modules based on XELOX response evaluation and ypStage. For the whole-genome chip data, WGCNA showed that only the magenta module was associated with XELOX response and ypStage (Fig. 2A). For miRNA chip data, the blue module was associated with XELOX response and negatively related to ypStage; however, the result was not significant (Fig. 2B). We then selected the magenta module for further Random forest model analysis. The results showed that the five most important genes were: 11757398_x_at (NORAD), 11760870_at, 11728805_a_at, 11728003_a_at and 11737881_x_at (Fig. 2C). Therefore, we further evaluated the role and mechanism involved in NORAD-mediated XELOX resistance. We used the online bioinformatics tool Starbase V3.0 to predict potential NORAD targets, and found that miR-433-3p was at the intersection of those targets with the blue module. TMT labeling was then used to compare the differential expression of related proteins in NORAD knockdown SGC-7901 cells and NC SGC-7901 cells. Following GO pathway analysis indicated that NORAD may have a role in autophagosome assembly, DNA repair, mitophagy, apoptotic process, double-strand break repair via homologous recombination, extracellular matrix organization, and et al. (Fig. 2D). Among the autophagy-related genes, we noticed that the expression of ATG5 and ATG12 were upregulated by NORAD based on the TMT-labeling method (Fig. 2E, F). Additionally, the TCGA and GTEx data also showed positive correlations between NORAD and ATG5 (Fig. 2G) and ATG12 (Fig. 2H).

In 379 gastric cancer patients, we found that NORAD expressed higher in gastric cancer patients compared with adjacent normal gastric tissues; and miR-433-3p expressed lower in gastric cancer tissues (Fig. 2I). Moreover, NORAD and miR-433-3p differentially expressed between recurrent and nonrecurrent gastric cancer patients (Fig. 2J). Furthermore, NORAD and miR-433-3p was correlated with overall survival and cancer-specific survival (Fig. 2K–N).

### NORAD was involved in regulating oxaliplatin resistance through mediating oxidative stress

**Fig. 3:**
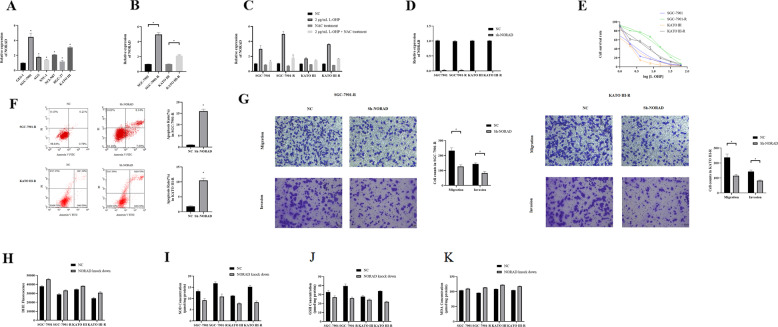
NORAD induced oxaliplatin resistance of gastric cancer cell. **A** qRT-PCR to test the expression of NORAD in gastric cancer cells compared with gastric normal cell, GES-1. One-way ANOVA test was used to test the difference of NORAD expression. GES-1 vs SGC-7901, *q* = 19.97, *P* < 0.000; GES-1 vs AGS, *q* = 4.28, *P* < 0.004; GES-1 vs SNU-1, *q* = 2.21, *P* = 0.174; GES-1 vs HGC-27, *q* = 6.35, *P* = 0.864; GES-1 vs KATO III, *q* = 0.916, *P* < 0.000. **B** qRT-PCR to test the expression of NORAD in SGC-7901-R (SGC-7901-R vs. SGC-7901, *t* = 28.20, *P* < 0.000) and KATO III-R (KATO III-R vs. KATO III, *t* = 18.51, *P* < 0.000) compared with parental cells. **C** qRT-PCR to test the expression of NORAD in SGC-7901-R and KATO III-R when treated with 2 μg/ml L-OHP (NC vs L-OHP: SGC-7901, *t* = 7.645, *P* = 0.001; SGC-7901-R, *t* = 20.08, *P* = 0.000;KATO III, *t* = 9.547, *P* = 0.001, KATO III-R, *t* = 48.13, *P* = 0.000. NC vs NAC: SGC-7901, *t* = 2.946, *P* = 0.011; SGC-7901-R, *t* = 6.752, *P* = 0.001; KATO III, *t* = 5.110, *P* = 0.002, KATO III-R, *t* = 20.87, *P* = 0.000. NC vs NAC + L-OHP: SGC-7901, *t* = 2.535, *P* = 0.032; SGC-7901-R, *t* = 2.868, *P* = 0.031; KATO III, *t* = 10.62, *P* = 0.000, KATO III-R, *t* = 26.71, *P* = 0.000). **D** NORAD expression after NORAD knockdown in SGC-7901, SGC-7901-R, KATO III and KATO III-R (SGC-7901, *t* = 60.73, *P* = 0.000; SGC-7901-R, *t* = 45.19, *P* = 0.000; KATO III, *t* = 119.1, *P* = 0.000; KATO III-R, *t* = 124.6, *P* = 0.000). **E** CCK-8 test for NORAD knockdown SGC-7901 and SGC-7901-R to evaluate the IC50 Tukey’s multiple comparisons test was used to evaluate the statistical significance of cell survival rate treated with different concentration of L-OHP. SGC-7901 vs SGC-7901-R: 0 μg/ml vs 1 μg/ml, *P* < 0.000; 0 μg/ml vs 1.5 μg/ml, *P* < 0.000; 0 μg/ml vs 2.0 μg/ml, *P* < 0.000; 0 μg/ml vs 2.5 μg/ml, *P* < 0.000. KATO III vs KATO III-R: 0 μg/ml vs 1 μg/ml, *P* < 0.000; 0 μg/ml vs 1.5 μg/ml, *P* < 0.000; 0 μg/ml vs 2.0 μg/ml, *P* < 0.000; 0 μg/ml vs 2.5 μg/ml, *P* < 0.000. **F** Flow cytometry apoptosis assay and the quantification result for NORAD knockdown in SGC-7901-R (*t* = 13.23, *P* = 0.000) and KATO III-R (*t* = 11.78, *P* = 0.000). **G** Transwell assay to evaluate the migration and invasion ability of NORAD knockdown in SGC-7901-R (Migration, *t* = 8.263, *P* = 0.002; Invasion, *t* = 8.046, *P* = 0.002) and KATO III-R (Migration, *t* = 8.695, *P* = 0.001; Invasion, *t* = 9.979, *P* = 0.001). **H** DHE method to test the ROS production in NORAD knockdown cells treated with 2 μg/ml L-OHP. (SGC-7901, *t* = 12.08, *P* = 0.001; SGC-7901-R, *t* = 6.191, *P* = 0.006; KATO III, *t* = 9.455, *P* = 0.002, KATO III-R, *t* = 6.379, *P* = 0.006) **I**–**K** SOD, GSH and MDA expression in NORAD knockdown cells when treated with 2 μg/ml L-OHP. 2I, SOD: SGC-7901, *t* = 4.067, *P* = 0.002; SGC-7901-R, *t* = 5.917, *P* = 0.002; KATO III, *t* = 15.87, *P* = 0.004, KATO III-R, *t* = 12.36, *P* = 0.001. 2J, GSH: SGC-7901, *t* = 4.228, *P* = 0.027; SGC-7901-R, *t* = 8.982, *P* = 0.002; KATO III, *t* = 3.830, *P* = 0.027, KATO III-R, *t* = 24.80, *P* = 0.000. 2K, MDA: SGC-7901, *t* = 5.364, *P* = 0.006; SGC-7901-R, *t* = 28.08, *P* = 0.000; KATO III, *t* = 16.61, *P* = 0.000, KATO III-R, *t* = 12.49, *P* = 0.000.

We found that NORAD was highly expressed in gastric cancer cell lines compared to normal gastric epithelium cell line, GES-1 (Fig. 3A), and that NORAD expression was higher in oxaliplatin-resistant cells than their parental cells (Fig. 3B). After treated with 2 μg/mL oxaliplatin, the expression of NORAD is significantly enhanced in gastric cancer cells (Fig. 3C). We then stably knocked down NORAD expression in both oxaliplatin-resistant cells and parental cells (Fig. 3D). NORAD knockdown led to reduced proliferation rates and IC50 values in SGC-7901 (IC50 = 2.24 μg/mL) and SGC-7901-R (IC50 = 11.68 μg/mL) cell lines; and KATO III (IC50 = 1.79 μg/mL) and KATO III-R (IC50 = 4.76 μg/mL) cells (Fig. 3E), indicative of a potential relationship between NORAD and oxaliplatin resistance. We also found that apoptosis could be induced by NORAD knockdown in SGC-7901-R and KATO III-R (Fig. 3F), suggesting that NORAD could induce oxaliplatin resistance. Further transwell assay showed that the migration and invasion ability of SGC-7901-R and KATO III-R was significantly decreased (Fig. 3G).

We then found that NORAD knockdown increased the ROS production and MDA level and decreased the SOD and GSH level in oxaliplatin-resistant cells and parental cells when treated with 2 μg/mL oxaliplatin (Fig. 3H–K).

### NORAD expression was induced by DNA damage repair response

**Fig. 4:**
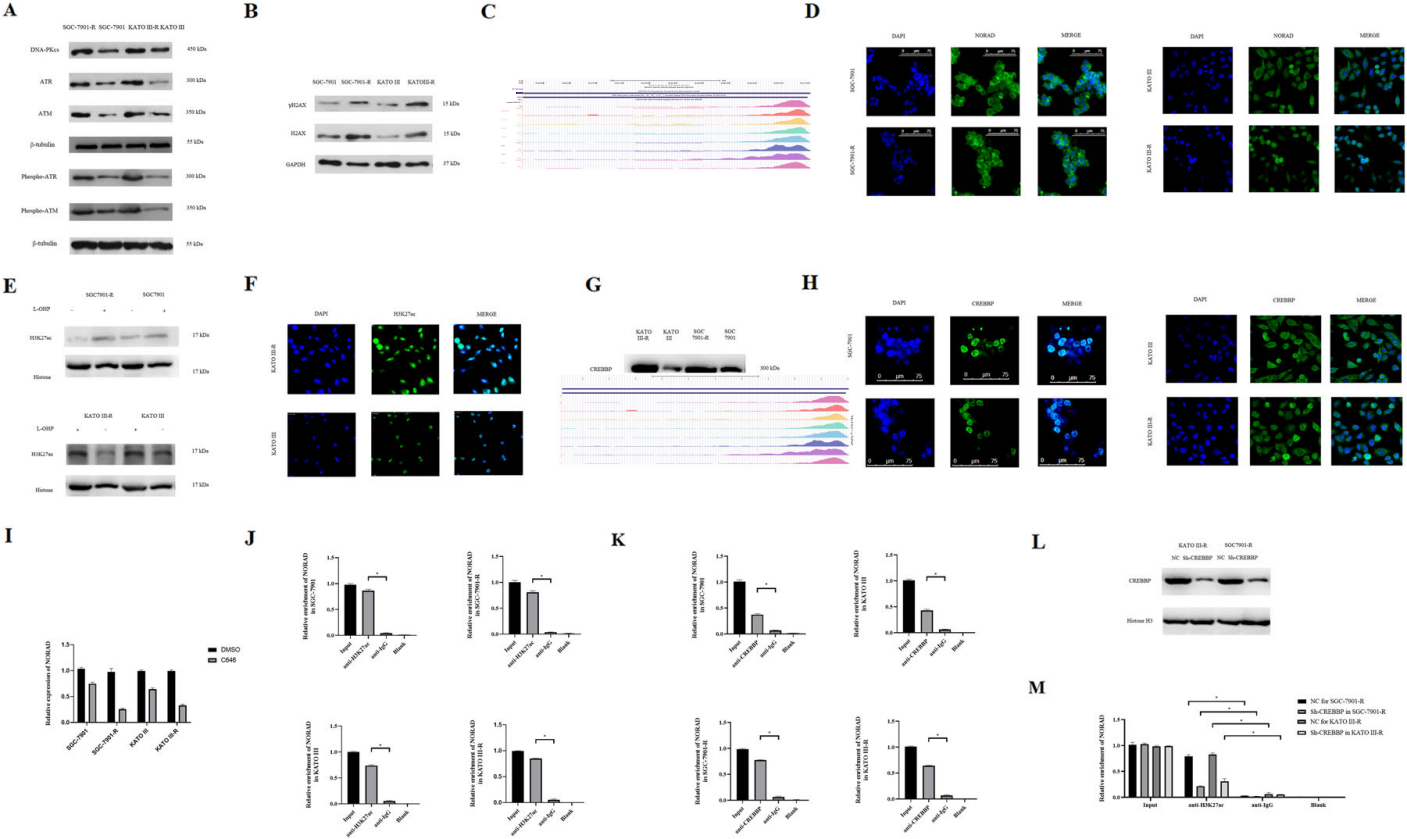
NORAD was associated with DNA damage repiar response and induced by H3K27ac. **A**, **B** Western blot to test the DNA damage repair related genes expression in SGC-7901 and KATO III: DNA-PKcs 450 kDa, ATR 300 kDa, Phospho-ATR (Ser428) 300 kDa, ATM 350kDA, Phospho-ATM (Ser1981) 350 kDa, H2AX 15 kDa, Phospho-Histone H2AX (Ser139) 15 kDa, β-Tubulin 55 kDa, GAPDH 37 kDa. **C** UCSC genome browser prediction about the enrichment of H3K27ac at the promoter region of NORAD in different cell lines. **D** NORAD location in SGC-7901, SGC-7901-R, KATO III, and KATO III-R by FISH tests. **E** H3K27ac expression in SGC-7901, SGC-7901-R, KATO III, and KATO III-R by western blot: H3K27ac 17 kDa, Histone H3 17 kDa. **F** H3K27ac location in SGC-7901, SGC-7901-R, KATO III, and KATO III-R by FISH tests. **G** CREBBP expression in SGC-7901, SGC-7901-R, KATO III, and KATO III-R by western blot: CREBBP 300 kDa, β-Actin 45 kDa. **H** CREBBP location in SGC-7901, SGC-7901-R, KATO III, and KATO III-R by FISH tests. **I** C646 can decrease the expression of NORAD in SGC-7901, SGC-7901-R, KATO III, and KATO III-R (SGC-7901, *t* = 11.52, *P* = 0.000; SGC-7901-R, *t* = 18.28, *P* = 0.000; KATO III, *t* = 17.64, *P* = 0.000, KATO III-R, *t* = 33.85, *P* = 0.000). **J** ChIP results to test the enrichment of H3K27ac at the promoter region of NORAD in SGC-7901, SGC-7901-R, KATO III, and KATO III-R. anti-H3k27ac vs anti IgG: SGC-7901, *t* = 17.19, *P* = 0.000; SGC-7901-R, *t* = 21.03, *P* = 0.000; KATO III, *t* = 31.22, *P* = 0.000, KATO III-R, *t* = 20.82, *P* = 0.000. **K** ChIP results to test the enrichment of CREBBP at the promoter region of NORAD in SGC-7901, SGC-7901-R, KATO III, and KATO III-R. anti-H3k27ac vs anti IgG: SGC-7901, *t* = 9.83, *P* = 0.000; SGC-7901-R, *t* = 15.83, *P* = 0.000; KATO III, *t* = 7.99, *P* = 0.000, KATO III-R, *t* = 19.92, *P* = 0.000. **L** Downregulation of CREBBP in SGC-7901-R and KATO III-R: CREBBP 300 kDa, β-Tubulin 55 kDa. **M** CREBBP knockdown can inhibit the enrichment of H3K27ac at the promoter region of NORAD in SGC-7901-R and KATO III-R. anti-H3k27ac: SGC-7901 vs SGC-7901-R, *t* = 16.23, *P* = 0.000; KATO III vs KATO III-R, *t* = 13.79, *P* = 0.000.

Western blot analysis indicated that DNA-PKcs, ATR, Phospho-ATR, ATM, Phospho-ATM, γH2AX, and H2AX were upregulated in oxaliplatin-resistant gastric cancer cells, indicating that DNA damage repair response was activated in SGC-7901-R and KATO III-R cells (Fig. 4A, B). Analysis using the UCSC genome browser indicated that H3K27ac enrichment might occur at the NORAD promoter, regulating its expression (Fig. 4C). The FISH results showed that NORAD was distributed in the nucleus and plasma in both oxaliplatin-resistant cells and their parental cells (Fig. 4D). After oxaliplatin treatment, the H3K27ac levels were induced in SGC-7901-R and KATO III-R cells, whereas in SGC-7901 and KATO III cells the levels of H3K27ac did not significantly increase (Fig. 4E). The FISH results showed that H3K27ac was predominantly distributed in the nucleus in oxaliplatin-resistant cells (Fig. 4F). Moreover, CREBBP (CREB-binding protein) expression was higher in oxaliplatin-resistant cells than that in parental cells (Fig. 4G). The FISH results demonstrated that CREBBP was mainly distributed in the nucleus in both oxaliplatin-resistant cells and parental cells (Fig. 4H).

However, when we treated the cells with C646, a CREBBP inhibitor, we found that NORAD expression was significantly decreased in both cell lines when compared with DMSO-treated controls (Fig. 4I). These results indicated that CREBBP-mediated regulation of H3K27ac may be important for the induction of NORAD expression. The ChIP assay showed that H3K27ac and CREBBP were enriched at the NORAD promoter of both gastric cancer cells and oxaliplatin-resistant cells (Fig. 4J, K). Furthermore, knockdown of CREBBP (Fig. 4L) reduced H3K27ac levels at the NORAD promoter in SGC-7901-R and KATO III-R cells (Fig. 4M). Combined, these results showed that the oxaliplatin-related DNA damage response can induce the binding of Histone H3 to CREBBP, thereby inducing NORAD expression.

### Autophagy was associated with oxidative stress and oxaliplatin resistance

**Fig. 5:**
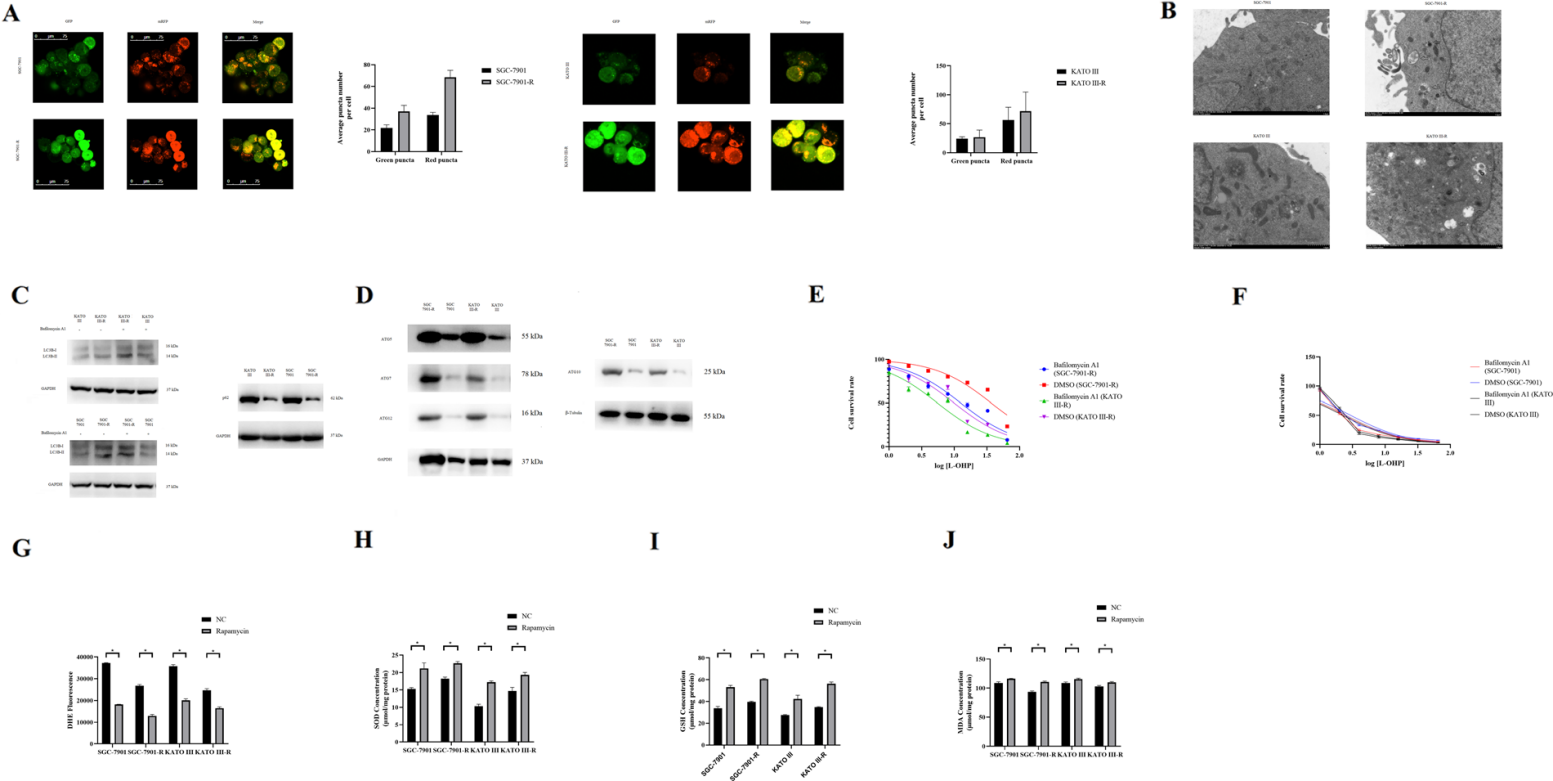
Autophagy mediated oxaliplatin resistance of gastric cancer cells. **A** Autophagy flux is enhanced in SGC-7901-R compared with SGC-7901; and KATO III-R compared with KATO III. The decreased green luciferase activity indicated enhanced autophagy flux: SGC-7901 vs SGC-7901-R, *t* = 3.893, *P* = 0.046; KATO III vs KATO III-R, *t* = 4.021, *P* = 0.039. **B** Electron microscope to observe autophagosomes in SGC-7901, SGC-7901-R, KATO III, and KATO III-R. **C** Expression of LC3B I/II and p62 in SGC-7901-R and KATO III-R compared with SGC-7901 and KATO III: LC3B I/II 16/14 kDa, p62 62 kDa, GAPDH 37 kDa. **D** Expression of autophagy associated genes in SGC-7901-R and KATO III-R compared with SGC-7901 and KATO III: ATG5(ATG5-ATG12 complex) 55 kDa, ATG12(free ATG12) 16 kDa, ATG7 78 kDa, GAPDH 37 kDa, ATG10 25 kDa, β-Tubulin 55 kDa. **E** The influence of Bafilomycin A1 on IC50 of SGC-7901-R and KATO III-R; DMSO is used as the control. Two way ANOVA test. SGC-7901-R: 1 μg/ml L-OHP, *t* = 9.496, *P* < 0.000; 2 μg/ml L-OHP, *t* = 12.48, *P* < 0.000, 4 μg/ml L-OHP, *t* = 18.89, *P* < 0.000; 8 μg/ml L-OHP, *t* = 21.23, *P* < 0.000; 16 μg/ml L-OHP, *t* = 27.21, *P* < 0.000; 32 μg/ml L-OHP, *t* = 25.50, *P* < 0.000; 64 μg/ml L-OHP, *t* = 16.64, *P* < 0.000. KATO III-R: 1 μg/ml L-OHP, *t* = 8.815, *P* < 0.000; 2 μg/ml L-OHP, *t* = 10.01, *P* < 0.000, 4 μg/ml L-OHP, *t* = 9.756, *P* < 0.000; 8 μg/ml L-OHP, *t* = 12.58, *P* < 0.000; 16 μg/ml L-OHP, *t* = 10.18, *P* < 0.000; 32 μg/ml L-OHP, *t* = 10.01, *P* < 0.000; 64 μg/ml L-OHP, *t* = 2.567, *P* < 0.085. **F** The influence of Bafilomycin A1 on IC50 of SGC-7901 and KATO III; DMSO is used as the control. Two way ANOVA test. SGC-7901: 1 μg/ml L-OHP, *t* = 0.1817, På 0.999; 2 μg/ml L-OHP, *t* = 7.207, *P* < 0.000, 4 μg/ml L-OHP, *t* = 12.48, *P* < 0.000; 8 μg/ml L-OHP, *t* = 10.81, *P* < 0.000; 16 μg/ml L-OHP, *t* = 2.256, *P* = 0.176, 32 μg/ml L-OHP, *t* = 2.998, *P* = 0.026; 64 μg/ml L-OHP, *t* = 3.391, *P* = 0.008. KATO III: 1 μg/ml L-OHP, *t* = 2.430, *P* = 0.117; 2 μg/ml L-OHP, *t* = 9.148, *P* < 0.000, 4 μg/ml L-OHP, *t* = 4.860, *P* < 0.000; 8 μg/ml L-OHP, *t* = 2.859, *P* = 0.038; 16 μg/ml L-OHP, *t* = 1.429, *P* = 0.698; 32 μg/ml L-OHP, *t* = 1.144, *P* = 0.875; 64 μg/ml L-OHP, *t* = 1.156, *P* = 0.843.**G** DHE method to test the ROS production in SGC-7901, SGC-7901-R, KATO III, and KATO III-R cell treated with rapamycin. (SGC-7901, *t* = 170.6, *P* = 0.000; SGC-7901-R, *t* = 26.87, *P* = 0.000; KATO III, *t* = 28.01, *P* = 0.000, KATO III-R, *t* = 15.74, *P* = 0.000). **H** SOD expression in SGC-7901, SGC-7901-R, KATO III, and KATO III-R when treated with rapamycin (SGC-7901, *t* = 6.352, *P* = 0.006; SGC-7901-R, *t* = 11.15, *P* = 0.001; KATO III, *t* = 18.37, *P* = 0.002, KATO III-R, *t* = 6.337, *P* = 0.006). **I** GSH expression in SGC-7901, SGC-7901-R, KATO III, and KATO III-R when treated with rapamycin (SGC-7901, *t* = 15.22, *P* = 0.000; SGC-7901-R, *t* = 40.78, *P* = 0.000; KATO III, *t* = 7.180, *P* = 0.002, KATO III-R, *t* = 21.58, *P* = 0.000). **J** MDA expression in SGC-7901, SGC-7901-R, KATO III, and KATO III-R when treated with rapamycin (SGC-7901, *t* = 5.295, *P* = 0.018; SGC-7901-R, *t* = 13.60, *P* = 0.001; KATO III, *t* = 4.850, *P* = 0.018, KATO III-R, *t* = 5.341, *P* = 0.018).

We found an enhanced autophagy flux in SGC-7901-R and KATO III-R cells (Fig. 5A). Further electron microscope was used to detect autophagosomes in oxaliplatin-resistant cells and their parental cells (Fig. 5B). In oxaliplatin-resistant cells, LC3B-II is highly expressed and p62 is lowly expressed compared with their parental cells (Fig. 5C). Furthermore, we found that ATG5, ATG12, ATG7, and ATG10 expression was increased in oxaliplatin-resistant cells compared with that in parental cells (Fig. 5D), indicating enhanced phagophore to autophagosome transformation.

We also found that Bafilomycin A1(10 nM for 18 h) could significantly decrease the IC50 of SGC-7901-R (IC50 = 12.55 μg/ml) and KATO III-R (IC50 = 5.52 μg/ml) cells compared with DMSO (SGC-7901-R,IC50 = 36.78 μg/ml; KATO III-R, IC50 = 9.95 μg/ml) (Fig. 5E), while only slightly influencing that of SGC-7901 (IC50 = 2.43 μg/ml) and KATO III cells (IC50 = 2.25 μg/ml) (Fig. 5F), thereby decreasing the RI (5.16) compared with DMSO group (RI = 12.35) and KATO III-R (RI = 2.16 vs DMSO group, RI = 4.41) as well.

Besides, we found that Rapamycin, an autophagy enhancer, reduced the ROS production and MDA level and increased the GSH and SOD level (Fig. 5G–J) when treated with 2 μg/ml oxaliplatin. We assumed that the activation of autophagy assists in DNA repair response and reducing DNA damage; thereby, reduced the oxidative stress and promoted oxaliplatin resistance.

### NORAD interacted with miR-433-3p to promote autophagy

**Fig. 6:**
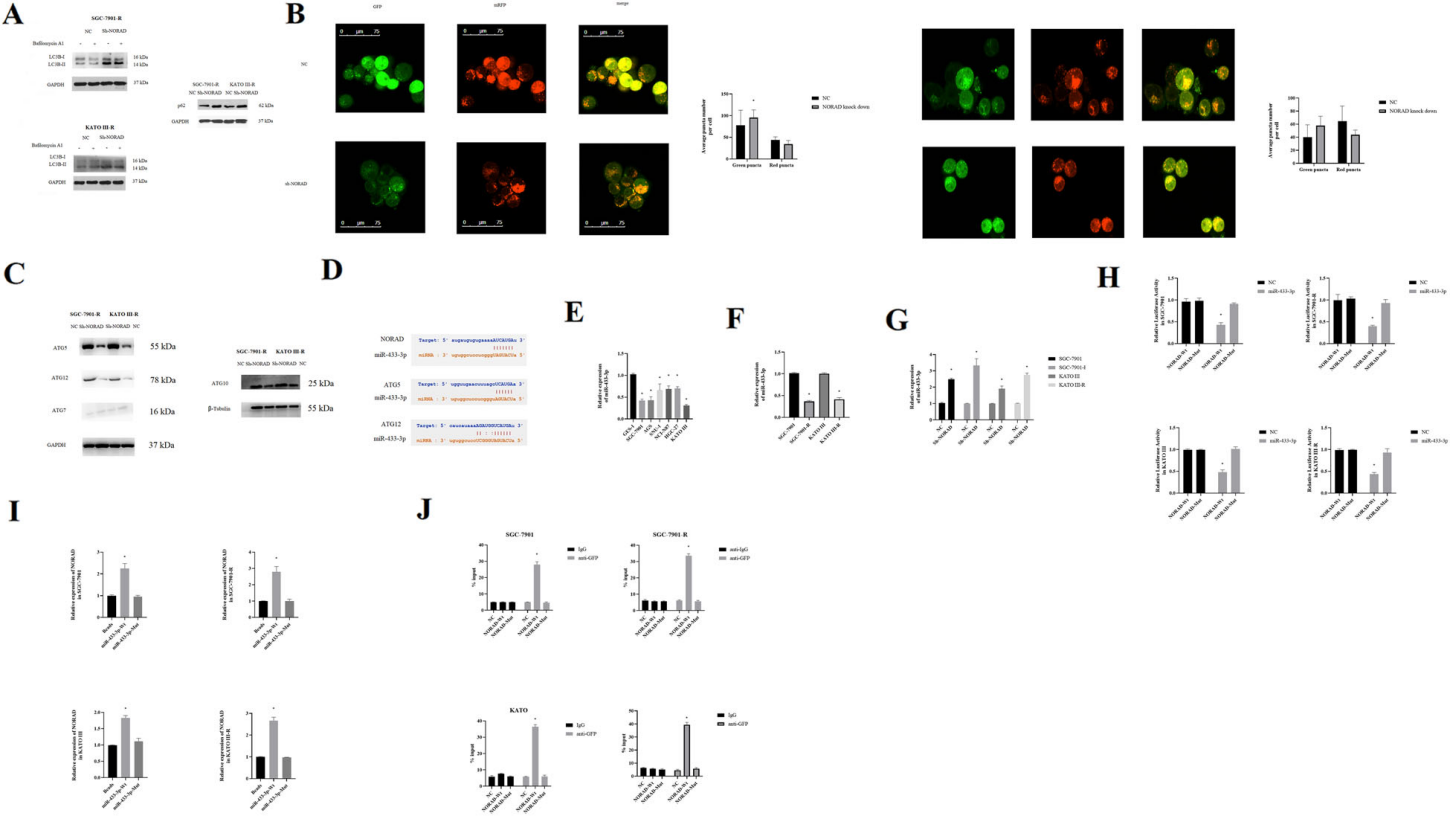
miR-433-3p was the downstream target of NORAD to regulate autophagy. **A** LC3B I/II expression in NORAD knockdown SGC-7901-R and KATO III-R cells: LC3B I/II 16/14 kDa, p62 62 kDa, GAPDH 37 kDa. **B** Autophagy flux was decreased by knocking down NORAD (SGC-7901-R, *t* = 4.241, *P* = 0.043; KATO III-R, *t* = 3.992, *P* = 0.036) **C** Expression of autophagy-related genes in NORAD knockdown SGC-7901-R and KATO III-R cell line: ATG5(ATG5-ATG12 complex) 55 kDa, ATG12(free ATG12) 16 kDa, ATG 78 kDa, GAPDH 37 kDa, ATG10 25 kDa, β-Tubulin 55 kDa. **D** Starbase prediction for the binding site of NORAD to miR-433-3p; and Starbase prediction for the binding site of miR-433-3p to ATG5 and ATG12. **E** qRT-PCR to detect the expression of miR-433-3p in gastric cancer compared with GES-1. One-way ANOVA: GES-1 vs SGC-7901, *q* = 10.37, *P* < 0.000; GES-1 vs AGS, *q* = 10.26, *P* < 0.000; GES-1 vs SNU-1, *q* = 6.274, *P* = 0.000; GES-1 vs HGC-27, *q* = 5.816, *P* = 0.000; GES-1 vs KATO III, *q* = 12.27, *P* < 0.000. **F** qRT-PCR to detect the expression of miR-433-3p in SGC-7901-R compared with SGC-7901 and KATO III-R compared with KATO III (SGC-7901 vs SGC-7901-R, *t* = 40.75, *P* = 0.000; KATO III vs KATO III-R, *t* = 23.00, *P* = 0.000). **G** qRT-PCR to detect the influence of NORAD knockdown on the expression of miR-433-3p in SGC-7901, SGC-7901-R, KATO III, and KATO III-R. (SGC-7901, *t* = 10.72, *P* < 0.000; SGC-7901-R, *t* = 17.31, *P* < 0.000; KATO III, *t* = 6.778, *P* < 0.000; KATO III-R, *t* = 12.98, *P* < 0.000). **H** Dual-luciferase reporter gene assay for NORAD and miR-433-3p (NORAD-Wt vs NORAD-Mut in miR-433-3p group: SGC-7901, *t* = 10.76, *P* < 0.000; SGC-7901-R, *t* = 7.986, *P* < 0.000; KATO III, *t* = 6.778, *P* < 0.000; KATO III-R, *t* = 12.98, *P* < 0.000). **I** RNA pull-down assay for the expression of NORAD in miR-433-3p-Wt and miR-433-3p-Mut group in SGC-7901, SGC-7901-R, KATO III, and KATO III-R (miR-433-3p-Wt vs miR-433-3p -Mut: SGC-7901, *t* = 9.321, *P* = 0.001; SGC-7901-R, *t* = 8.962, *P* = 0.001; KATO III, *t* = 10.60, *P* = 0.000; KATO III-R, *t* = 19.07, *P* = 0.000). **J** RIP assay to detect the enrichment of miR-433-3p (NORAD-Wt vs NORAD-Mut: SGC-7901, *t* = 32.111, *P* = 0.000; SGC-7901-R, *t* = 15.12, *P* = 0.000; KATO III, *t* = 24.21, *P* = 0.000; KATO III-R, *t* = 13.22, *P* = 0.000).

When we knocked down NORAD in SGC-7901-R and KATO III-R cells, we found that LC3B-I expression was increased, and LC3B-II expression was inhibited, indicating that the autophagy flux was inhibited (Fig. 6A). Moreover, autophagy flux inhibition was detected by knocking down NORAD (Fig. 6B). We then knocked down NORAD expression in SGC-7901-R, and observed that ATG5 and ATG12 expression was inhibited; however, the expression of ATG7 and ATG10 remained unchanged (Fig. 6C). Based on Starbase V3.0, we predicted that miR-433-3p might be a target of NORAD (Fig. 6D).

We found that miR-433-3p expressed lowly in gastric cancer cells (Fig. 6E) and its expression was decreased in SGC-7901-R and KATO III-R cell line (Fig. 6F). miR-433-3p expression was increased in NORAD knockdown gastric cancer cells (Fig. 6G).

In the dual-luciferase reporter assay, the relative luciferase activity was significantly decreased in the NORAD-WT group in both SGC-7901 and SGC-7901-R cells (Fig. 6H), indicating that NORAG directly interacts with miR-433-3p.

The RNA pull-down assay indicated that the expression of NORAD was higher in the miR-433-3p-Wt group compared with that in the miR-433-3p-Mut group in SGC-7901 and SGC-7901-R cells (Fig. 6I).

We then mutated NORAD for the RIP assay. The results showed that miR-433-3p was enriched in the NORAD-Wt group compared with that in the NORAD-Mut group (Fig. 6J).

Taken together, these results indicated that NORAD knockdown inhibited autophagy by decreasing the expression of miR-433-3p.

### MiR-433-3p downregulated the expression of ATG5 and ATG12

**Fig. 7:**
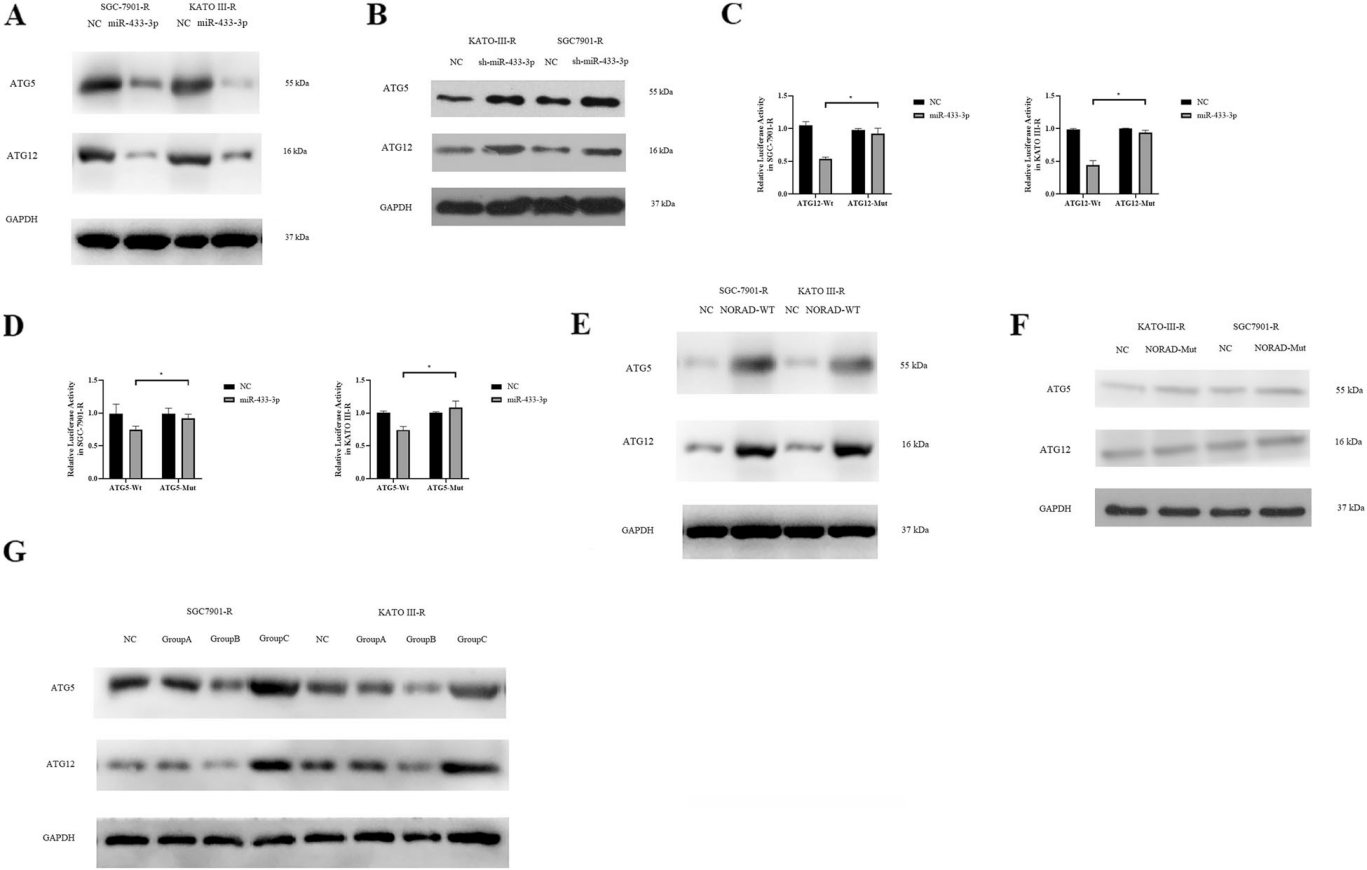
miR-433-3p regulated autophagy by targeting ATG5 and ATG12. **A** Expression of ATG5 and ATG12 in miR-433-3p upregulated SGC-7901-R and KATO III-R cell line: ATG5(ATG5-ATG12 complex) 55 kDa, ATG12(free ATG12) 16 kDa, GAPDH 37 kDa. **B** Expression of ATG5 and ATG12 in miR-433-3p knockdown SGC-7901-R and KATO III-R cell line: ATG5(ATG5-ATG12 complex) 55 kDa, ATG12(free ATG12) 16 kDa, ATG 78 kDa, GAPDH 37 kDa. **C** Dual-luciferase reporter gene assay for miR-433-3p and ATG12 in SGC-7901-R (*t* = 8.789, *P* < 0.000) and KATO III-R (*t* = 15.29, *P* < 0.000). **D** Dual-luciferase reporter gene assay for miR-433-3p and ATG5 in SGC-7901-R (*t* = 2.260, *P* = 0.105) and KATO III-R (*t* = 7.296, *P* = 0.002). **E** Expression of ATG5 and ATG12 in NORAD-Wt(containing the binding site for miR-433-3p) upregulated SGC-7901-R and KATO III-R cell line: ATG5(ATG5-ATG12 complex) 55 kDa, ATG12(free ATG12) 16 kDa, ATG 78 kDa, GAPDH 37 kDa. **F** Expression of ATG5 and ATG12 in NORAD-Mut (containing the binding site for miR-433-3p) upregulated SGC-7901-R and KATO III-R cell line: ATG5(ATG5-ATG12 complex) 55 kDa, ATG12(free ATG12) 16 kDa, ATG 78 kDa, GAPDH 37 kDa. **G** miR-433-3p knockdown could reverse the downregulation of ATG12 and ATG5 that was induced by NORAD knockdown. Group A: NORAD knockdown + miR-433-3p knockdown; Group B: NORAD knockdown; Group C: miR-433-3p knockdown.

When we overexpressed miR-433-3p in SGC-7901-R and KATO III-R, the expression of ATG5 and ATG12 was reduced (Fig. 7A). However, knockdown of miR-433-3p did not lead to a significant increase in the expression of ATG5 or ATG12 in SGC-7901-R (Fig. 7B). The dual-luciferase reporter assay showed that miR-433-3p directly interacted with the 3′-UTR of *ATG12* (Fig. 7C), and the relative luciferase activity was also reduced in *ATG5* wild-type cells in SGC-7901-R and KATO III-R (Fig. 7D). Moreover, we found that overexpression of NORAD-WT induced the expression of ATG12 and ATG5 (Fig. 7E), whereas overexpression of NORAD-Mut did not (Fig. 7F). Furthermore, miR-433-3p knockdown could reverse the downregulation of ATG12 and ATG5 that was induced by NORAD knockdown (Fig. 7G).

### NORAD knockdown mediated oxidative stress and inhibited oxaliplatin resistance in vivo

**Fig. 8:**
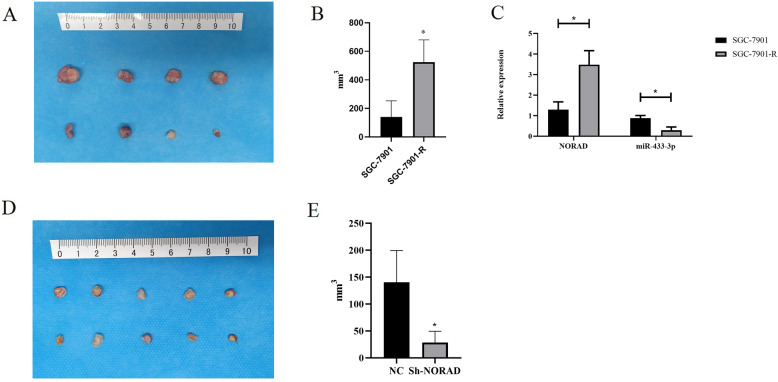
NORAD regulated oxaliplatin resistance in vivo. **A** Xenograft tumors from 10 nude mice’ right flank. SGC-7901 and SGC-7901-R was used to produce the xenograft tumors. **B** Estimated volumes of xenograft tumors: volume = π/6 × width^2^ × length (*t* = 4.383, *P* = 0.002). **C** NORAD (*t* = 14.52, *P* = 0.001) and miR-433-3p (*t* = 4.98, *P* = 0.031) expression in SGC-7901 and SGC-7901-R generated xenograft tumors. **D** Xenograft tumors from 10 nude mice’ right flank. SGC-7901-R transduced with NORAD knockdown lentivirus and relevant NC cells were used to produce the xenograft tumors. **E** Estimated volumes of xenograft tumors: volume = π/6 × width^2^ × length (*t* = 5.489, *P* = 0.007).

We have grafted SGC-7901 and SGC-7901-R cells into nude mice, who were subjected to 10 μg/kg oxaliplatin every week for 2 weeks. Then tumors were harvested (Fig. 8A) and the quantified estimated tumor burden results showed that SGC-7901-R mice can well tolerant oxaliplatin (Fig. 8B).

Then, mice, who were subjected to 10 μg/kg oxaliplatin every week for 2 weeks, were grated with NORAD knockdown and relevant NC SGC-7901-R cell line. Tumors were isolated after the same treatment stated above (Fig. 8C). The estimated tumor volume result indicated that NORAD knockdown can significantly inhibit the cell tolerance to oxaliplatin (Fig. 8D).

## Discussion

Oxaliplatin is a third-generation, platinum-containing anticancer agent, which exerts its anticancer effects through the induction of DNA damage and consequent inhibition of DNA replication^30^. Oxaliplatin is well known for its greater efficacy in controlling cancer foci and inducing fewer side effects compared with other platinum-based drugs^30^. The employment of an oxaliplatin-based treatment strategy, such as postoperative XELOX therapy, has led to a significant improvement in the overall survival and reduction in the local recurrence rate of gastrointestinal cancers, especially gastric cancer^31^. However, some patients will experience oxaliplatin resistance and a dramatic reduction in the effectiveness of the drug, which may be due to continuous exposure to oxaliplatin^32^. Oxaliplatin resistance always leads to the failure of chemotherapy and results in recurrence and worse prognosis^33^. Therefore, it is vital that the molecular mechanisms involved in oxaliplatin resistance are clarified, thereby laying the foundation for the identification of novel therapeutic strategies.

In this manuscript, we found that oxidative stress, which can be induced by oxaliplatin treatment, is an important regulator in mediating oxaliplatin resistance. Furthermore, we noticed that SGC-7901-R has the ability to suppress oxidative stress. NORAD knockdown can abrogate the inhibiting ability of SGC-7901-R in ROS production. Therefore, we preliminary hypothesized that NORAD is overexpressed in response to ROS production and thus acted as an oxidative stress suppressor. Subsequently, we identified NORAD as a key regulator of oxaliplatin resistance. Our group previously reported the significant role of NORAD in mediating radiation resistance through the promotion of the DNA damage repair response^19^. In this manuscript, we have identified the enhanced DNA damage response in SGC-7901-R. We assumed that NORAD is induced by DNA damage and promotes DNA damage response; the continuous activation of NORAD is essential for SGC-7901-R to suppress ROS production and resist oxidative stress.

Previously, a plethora of studies have presented that oxidative stress can induce the autophagy flux. We found that autophagy enhancer, rapamycin, can decrease the ROS production, indicating the important role of autophagy in regulating oxidative stress of SGC-7901-R cell.

Various studies have shown that activation of autophagy is associated with oxaliplatin resistance^9,10,13,14^. Here, we observed that autophagy flux was enhanced in oxaliplatin-resistant cells. The TMT-labeling result indicated that NORAD may promote autophagy by stabilizing the formation of the ATG5-ATG12 complex. NORAD knockdown inhibited the autophagy flux and significantly decreased the IC50 and RI of SGC-7901-R cells. This strongly suggested that autophagy is involved in oxaliplatin resistance in gastric cancer and inhibiting autophagy by targeting NORAD may be a potential strategy to reverse the oxaliplatin-resistant status of SGC-7901-R cells. Further experiments showed that NORAD did not affect the activity of ATG10 and ATG7, essential mediators of ATG5-ATG12 conjugation^23^. Bioinformatic analysis identified that miR-433-3p binds to the 3′-UTR of *ATG5* and *ATG12*, which was further confirmed by dual-luciferase reporter assays. Taken together, we have shown that NORAD sponges miR-433-3p, which upregulates the expression of *ATG5* and *ATG12*, thereby enhancing the autophagy flux.

As previously stated, oxaliplatin mainly causes DNA damage to exert its anticancer effects, indicating that activation of the DNA damage repair response is largely involved in mediating oxaliplatin resistance^7^. In this study, we observed that DNA damage repair was constitutively activated in oxaliplatin-resistant cells. Furthermore, we also found aberrantly high levels of H3K27ac, a form of histone acetylation mainly found in the promoter region of specific genes in oxaliplatin-resistant cells that promotes their expression^34^. As we found that H3K27ac is significantly enriched in the promoter region of NORAD, as assessed through UCSC prediction and ChIP results, we think that the oxaliplatin-mediated DNA damage repair response may induce the expression of NORAD to exert its regulatory function in oxaliplatin resistance by facilitating H3K27ac.

Accumulating evidence supports that diverse noncoding RNAs, including lncRNAs and miRNAs, have crucial biological functions in drug resistance^35^. For example, MALAT1 sponges miR-23b-3p to promote autophagy-related chemoresistance, giving rise to the vincristine resistance^36^. Moreover, the RNA gene *TINCR* can promote EMT through competing for the binding site of miR-125b on *HER2*, resulting in the trastuzumab resistance^37^. The FISH results indicated that NORAD is primarily localized in the cytoplasm, suggesting that NORAD may act as a sponge and compete with miRNAs for the binding to target gene promoters. Based on the miRNA chip and bioinformatic analyses, we propose that NORAD may act as a molecular sponge for miR-433-3p.

## Conclusions

Graphical In conclusion, oxidative stress is enhanced by treating with oxaliplatin. NORAD knockdown induced oxidative stress by impairing SOD and GSH expression in SGC-7901-R. Then, oxaliplatin-induced DNA damage repair response can induce H3K27ac and activate CREBBP, which are enriched at the promoter region of NORAD, thereby upregulating its expression. NORAD will then enhance the autophagy flux by stabilizing ATG5-ATG12 conjugation through sponging miR-433-3p, leading to enhanced oxidative stress, and finally resulting in oxaliplatin resistance. Targeting NORAD can reverse the oxaliplatin-resistant status of SGC-7901-R cells, suggesting that targeting NORAD may be a novel therapeutic strategy for suppressing oxaliplatin resistance.

## Supplementary information

Supplementary Figure legends

Supplementary Figure1

Supplementary Figure2

Supplementary Figure3

Supplementary Figure4

Supplementary Figure5

Supplementary Figure6

## Data Availability

Data would be made available on request.

## Materials availability

Materials would be made available on request.
