# Supplementary material for: Oxidative stress activates NORAD expression by H3K27ac and promotes oxaliplatin resistance in gastric cancer by enhancing autophagy flux via targeting the miR-433-3p
Source: Cell Death Dis. 2021 Jan 18;12(1):90. doi: 10.1038/s41419-020-03368-y (PMC7814071; doi:10.1038/s41419-020-03368-y)
Supplement: Supplementary file 1 — Supplementary Figure legends [file 41419_2020_3368_MOESM1_ESM.docx]

Sup 1 **A** Quantification for figure 4A and 4B. **B** Quantification for figure 4E. **C** Quantification for figure 4G. **D** Quantification for figure 4L. **E** Quantification for Figure 5C. **F** Quantification for Figure 5D. **G** Quantification for Figure 6A.

Sup 2 **A** Quantification for Figure 7A. **B** Quantification for Figure 7B. **C** Quantification for Figure 7E. **D** Quantification for Figure 7F. **E** Quantification for Figure 7G. **F** Quantification for Figure 7G.

Sup 3 **A** Repeated western blot for Figure 4A. **B** Repeated western blot for Figure 4B. **C** Repeated western blot for Figure 4E. **D** Repeated western blot for Figure 4G. **E** Repeated western blot for Figure 4L.

Sup 4 **A** Repeated western blot for Figure 5C. **B** Repeated western blot for Figure 5D.

Sup 5 **A** Repeated western blot for Figure 6A. **B** Repeated western blot for Figure 6C.

Sup6 **A** Repeated western blot for Figure 7A. **B** Repeated western blot for Figure 7B. **C** Repeated western blot for Figure 7E. **D** Repeated western blot for Figure 7F. **E** Repeated western blot for Figure 7G.
