# Supplementary figures and images for: Oxidative stress activates NORAD expression by H3K27ac and promotes oxaliplatin resistance in gastric cancer by enhancing autophagy flux via targeting the miR-433-3p
Source: Cell Death Dis. 2021 Jan 18;12(1):90. doi: 10.1038/s41419-020-03368-y (PMC7814071; doi:10.1038/s41419-020-03368-y)

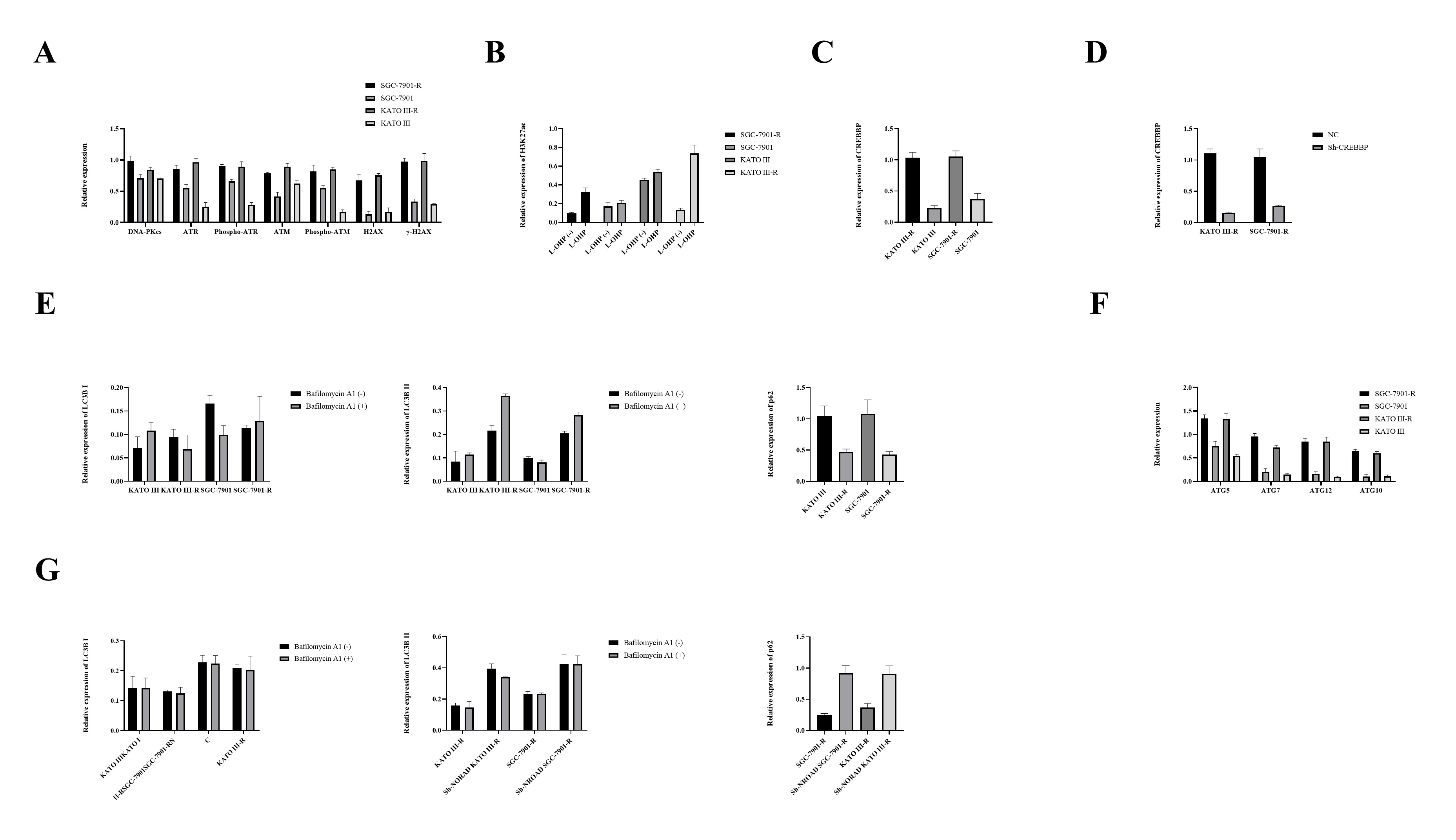

Supplement: Supplementary file 2 — Supplementary Figure1 [file 41419_2020_3368_MOESM2_ESM.jpg]

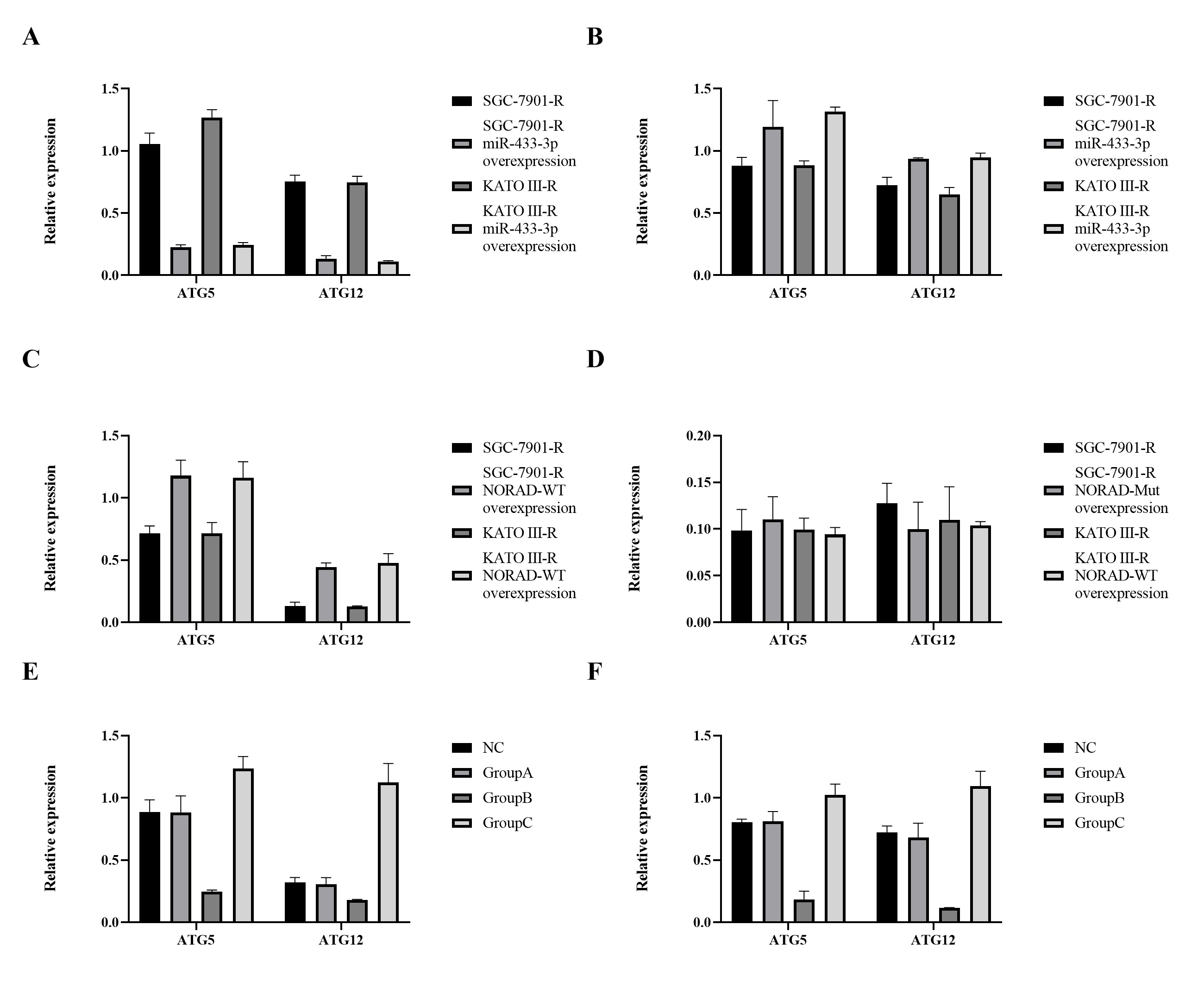

Supplement: Supplementary file 3 — Supplementary Figure2 [file 41419_2020_3368_MOESM3_ESM.jpg]

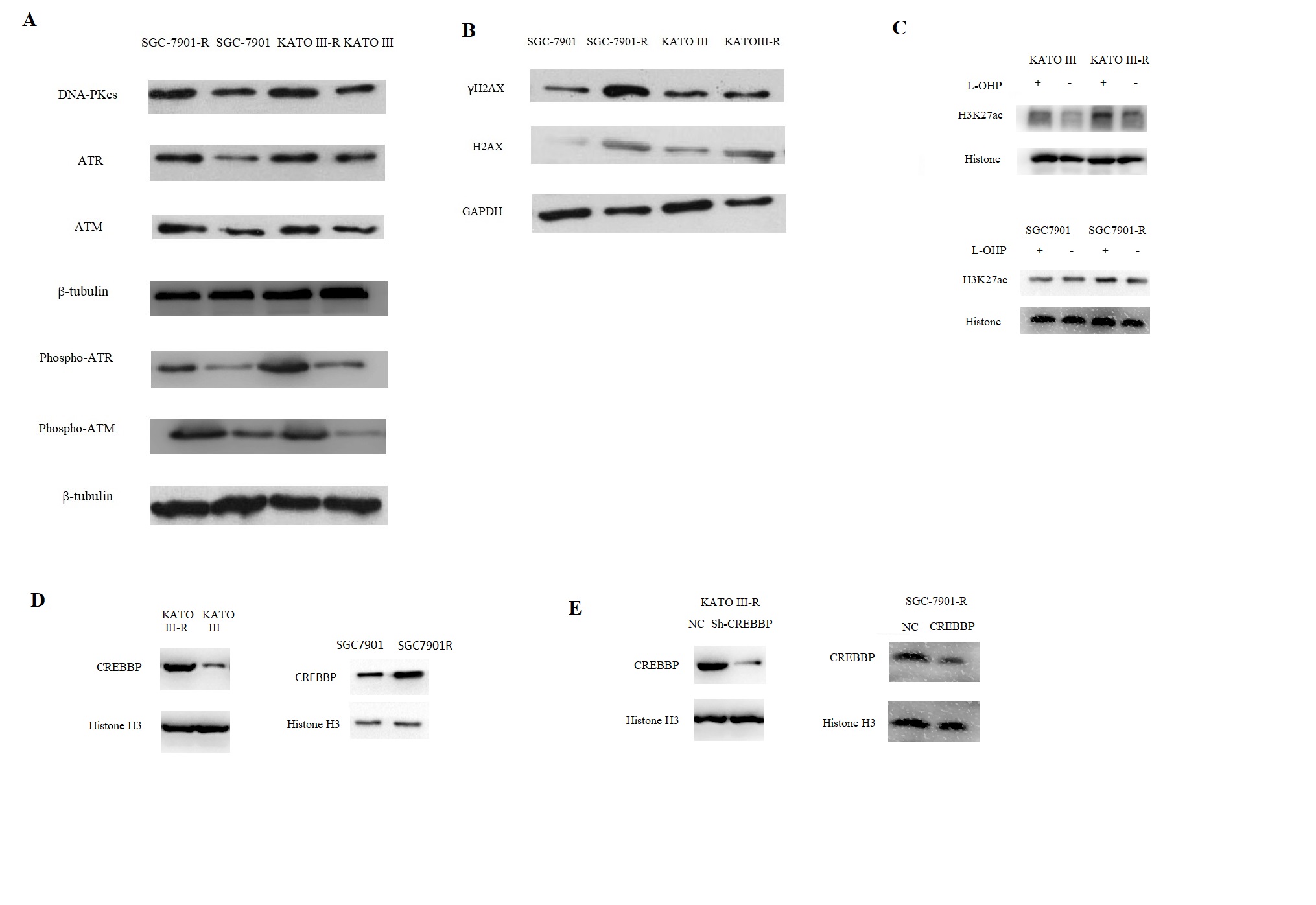

Supplement: Supplementary file 4 — Supplementary Figure3 [file 41419_2020_3368_MOESM4_ESM.jpg]

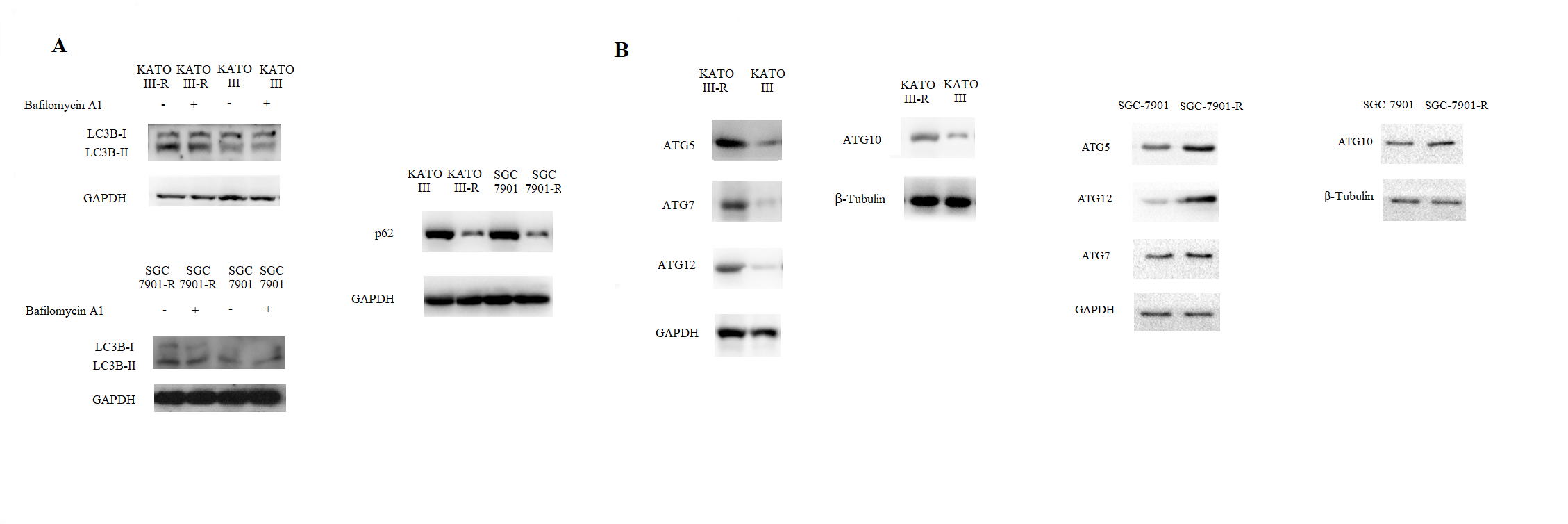

Supplement: Supplementary file 5 — Supplementary Figure4 [file 41419_2020_3368_MOESM5_ESM.tif]

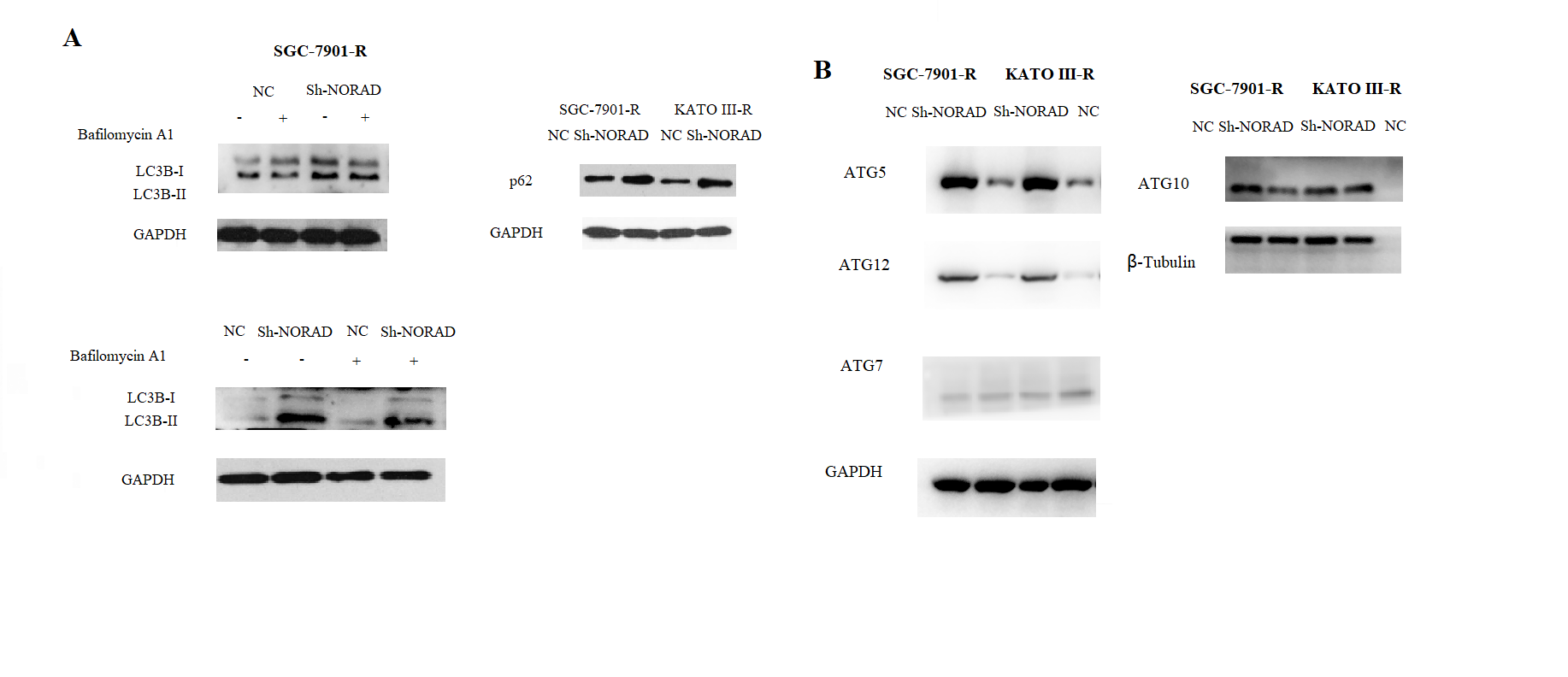

Supplement: Supplementary file 6 — Supplementary Figure5 [file 41419_2020_3368_MOESM6_ESM.tif]

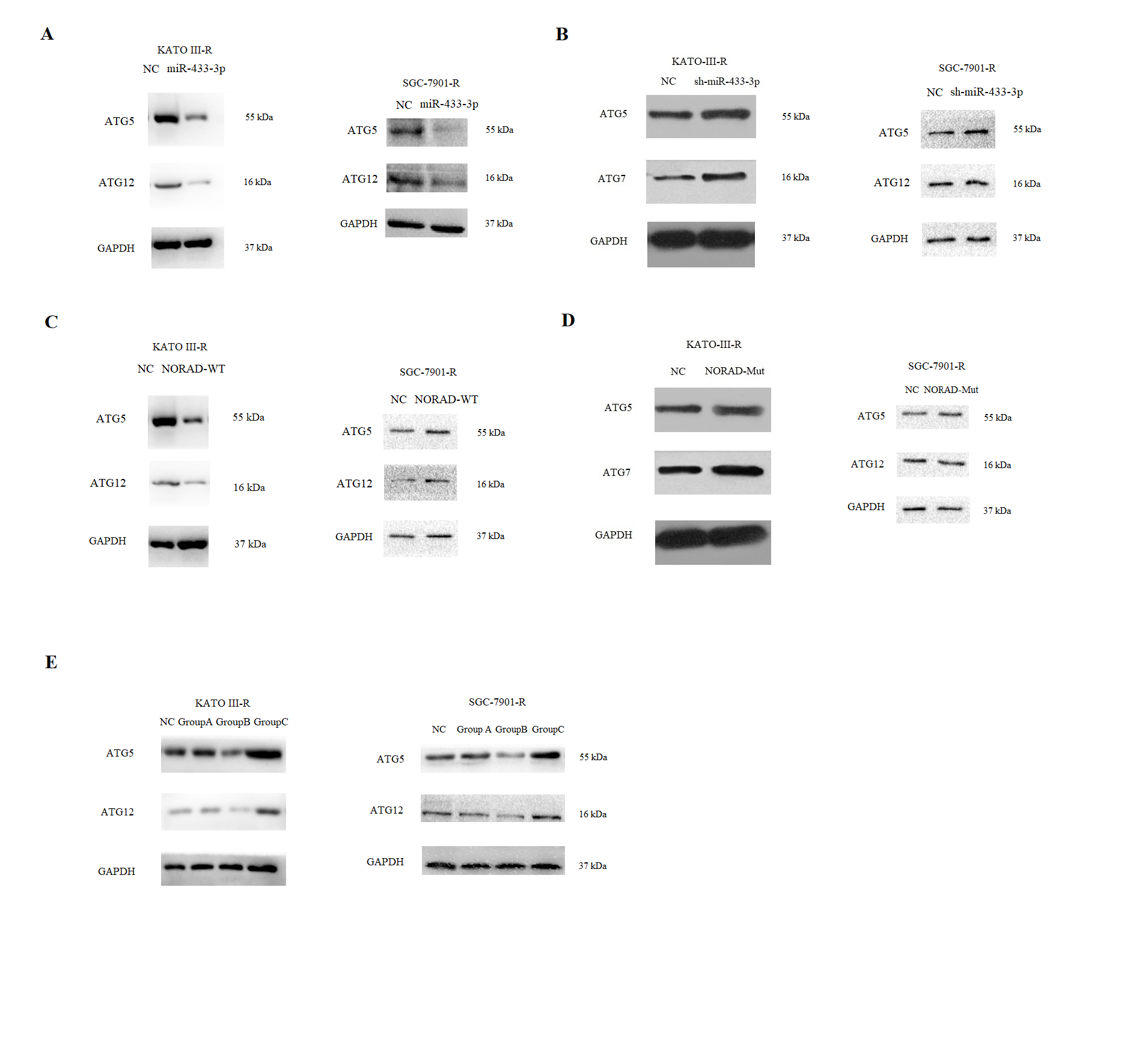

Supplement: Supplementary file 7 — Supplementary Figure6 [file 41419_2020_3368_MOESM7_ESM.tif]
